# Neurocognitive functioning and health-related quality of life of children after pediatric intensive care admission: a systematic review

**DOI:** 10.1007/s11136-022-03124-z

**Published:** 2022-03-31

**Authors:** José A. Hordijk, Sascha C. Verbruggen, Corinne M. Buysse, Elisabeth M. Utens, Koen F. Joosten, Karolijn Dulfer

**Affiliations:** 1 Intensive Care, Department of Pediatrics and Pediatric Surgery, Erasmus MC - Sophia Children’s Hospital, Dr. Molewaterplein 60, 3015 GJ Rotterdam, The Netherlands; 2 Research Institute of Child Development and Education, University of Amsterdam, Nieuwe Achtergracht 127, 1018 WS Amsterdam, The Netherlands; 3 Academic Center for Child Psychiatry the Bascule/Department of Child and Adolescent Psychiatry, Academic Medical Center, Rijksstraatweg 145, 1115 AP Amsterdam, The Netherlands; 4 Department of Child and Adolescent Psychiatry/Psychology, Erasmus MC - Sophia Children’s Hospital, Wytemaweg 8, 3015 CN Rotterdam, The Netherlands

**Keywords:** Pediatric intensive care unit, Critical illness, Child health, Neuropsychology, Quality of life, Follow-up studies

## Abstract

**Objective:**

This study systematically reviewed recent findings on neurocognitive functioning and health-related quality of life (HRQoL) of children after pediatric intensive care unit admission (PICU).

**Data sources:**

Electronic databases searched included Embase, Medline Ovid, Web of Science, Cochrane CENTRAL, and Google Scholar. The search was limited to studies published in the last five years (2015–2019).

**Study selection:**

Original studies assessing neurocognitive functioning or HRQoL in children who were previously admitted to the PICU were included in this systematic review.

**Data extraction:**

Of the 3649 identified studies, 299 met the inclusion criteria based on title abstract screening. After full-text screening, 75 articles were included in the qualitative data reviewing: 38 on neurocognitive functioning, 33 on HRQoL, and 4 on both outcomes.

**Data synthesis:**

Studies examining neurocognitive functioning found overall worse scores for general intellectual functioning, attention, processing speed, memory, and executive functioning. Studies investigating HRQoL found overall worse scores for both physical and psychosocial HRQoL. On the short term (≤ 12 months), most studies reported HRQoL impairments, whereas in some long-term studies HRQoL normalized. The effectiveness of the few intervention studies during and after PICU admission on long-term outcomes varied.

**Conclusions:**

PICU survivors have lower scores for neurocognitive functioning and HRQoL than children from the general population. A structured follow-up program after a PICU admission is needed to identify those children and parents who are at risk. However, more research is needed into testing interventions in randomized controlled trials aiming on preventing or improving impairments in critically ill children during and after PICU admission.

**Supplementary Information:**

The online version contains supplementary material available at 10.1007/s11136-022-03124-z.

## Plain English summary

As a result of improved survival rates, the focus of research into pediatric intensive care patients has shifted from mortality to morbidities after discharge. Most studies focusing on psychosocial follow-up outcomes investigate neurocognitive functioning and health-related quality of life (HRQoL). The key problem is that no overview of the most important psychosocial outcomes after pediatric intensive care unit (PICU) admission exists in the literature. The current study systematically reviews recent findings regarding neurocognitive and health-related quality of life outcomes after pediatric critical illness. Furthermore, it also gives information about interventions that were conducted to improve these outcomes and gives implications for a structured follow-up after PICU admission. Studies found overall worse scores for general intellectual functioning, attention, processing speed, memory, and executive functioning. Studies investigating HRQoL found overall worse scores for both physical and psychosocial HRQoL.

## Introduction

Medical and technological improvements in the pediatric intensive care unit (PICU) in the past decades have reduced mortality dramatically to approximately 2–3% [1]. The focus in research has therefore shifted from mortality towards ongoing morbidities and daily life outcomes for critically ill children and their families after PICU discharge. Critical illness in children occurs at a time of growth and development and may therefore influence maturation trajectories in multiple domains [1]. This has recently been conceptualized as the internationally acknowledged post-intensive care syndrome in children (PICS-p) [2]. PICS-p covers morbidities in the following four developmental domains: physical, emotional, social, and cognitive functioning. Children and their families have to adapt to the changes in these PICS-p domains in reaching a new normal in daily life. The subjective evaluation of the physical, emotional, and social domains together is conceptualized as health-related quality of life (HRQoL) [3]. The fourth domain, cognitive functioning refers to the internal mental processes underlying how children perceive stimuli, remember, speak, think, make decisions, and solve problems [4]. The four domains could be assessed both subjectively and objectively and give a complete view of the child’s daily functioning. Other earlier reviews in the field of PICS-p after PICU admission were done in specific PICU sub-population [5], excluded specific disease processes, interventions or age-groups [5–7], or under-recognized the importance of HRQoL as embedded assessment of social and emotional outcomes in validating the PICS-p framework [8, 9].

Our present review includes all recent studies until 2021 investigating all four PICS-p domains: physical, emotional, and social functioning (HRQoL) and cognitive functioning in the total, heterogeneous PICU population from 0 to 17 years old. This review will give an integrated view of the current knowledge of important PICS-p outcomes after pediatric critical illness and its predictors. Moreover, it also discusses the results of randomized controlled trials (RCTs) aiming on modifying PICS-p outcomes and implications for clinical follow-up programs.

## Materials and methods

### Search strategy and selection criteria

The systematic literature search was conducted on February 26, 2021 to identify relevant articles published 2015–2020 in the following electronic databases: Embase, Medline Ovid, Web of Science, Cochrane CENTRAL, and Google Scholar. Keywords used were pediatric intensive care, children, adolescents, neurocognitive functioning, and health-related quality of life (see Online Resource 1a for complete search strategy). The articles that resulted from the search were independently screened on title and abstract by two reviewers (JH + KD). Abstracts that did not overlap were discussed (JH + KD). Full-text screening was conducted by one reviewer (JH) and discussed with the other reviewer (KD). When full-text inclusion was debatable, another reviewer made the final decision about inclusion (KJ).

Studies were included when the following eligibility criteria were met: term born children to 18 years old who were admitted to a PICU; (neuro)cognitive functioning and/or HRQoL assessed with validated, age appropriate measurements with country-specific normative data or control groups in children who survived a PICU admission.

Exclusion criteria were the following: case reports and editorials; studies published in other languages than English; studies including neonates born preterm (gestational age < 36 weeks) admitted to the PICU or NICU when not reporting outcomes of term born children separately. When it was not clear whether a study included children born preterm (< 36 weeks), the authors were approached by e-mail to check. In case there was no response or when it remained unclear, the study was excluded. With regard to the neurocognitive outcome measurements, studies assessing neurocognitive functioning both by clinical and/or by proxy-reports of the parents or caregivers were included.

### Data extraction

For all eligible studies, the following variables were extracted: population studied, age at PICU admission (or age at follow-up in case age at admission was not mentioned), sample size, study design, time between PICU admission and follow-up, measurements used (clinical tests, parent-reported or self-reported questionnaires), and main results. As to executing a meta-analysis of the results, this is discouraged based on the following conditions: both RCTs and observational studies are included in this systematic review, different approaches for analyses were used in studies involved, and different outcomes with different instruments were assessed [10, 11]. Under such conditions, a meta-analysis will not lead to a meaningful interpretation and was therefore not performed. A qualitative synthesis of the main results was done as follows: when the group mean of the patients was more than 1SD below normative data or mean score of healthy control children, scores were judged as impaired, when the group mean of patients was lower than normative data/healthy control data but within the normal range (within 1SD below the norm to average), they were judged as lower than average, and when scores were comparable or higher than the norm/healthy control data, they were judged as not impaired (see supplemental Table 1a and Online Resource 1b). To present the results in a structured way, studies in infants (group mean or median age less than 12 months) and children (group mean/median age 12 months or older) were separately reported as well as studies focusing on the short term (12 months or less) and studies focusing on the long term (more than 12 months) after PICU admission. The qualitative synthesis of HRQoL is divided into overall HRQoL, physical HRQoL, and psychosocial HRQoL, following the scale structure of most HRQoL instruments. Both domains emotional functioning and social functioning are represented in the psychosocial HRQoL scale. When emotional and/or social subscales were reported separately, we averaged the outcomes. Normative data or control group outcomes reported in the paper were used as references. When these were not mentioned, internationally accepted normative data were used as reference.

**Table 1:**
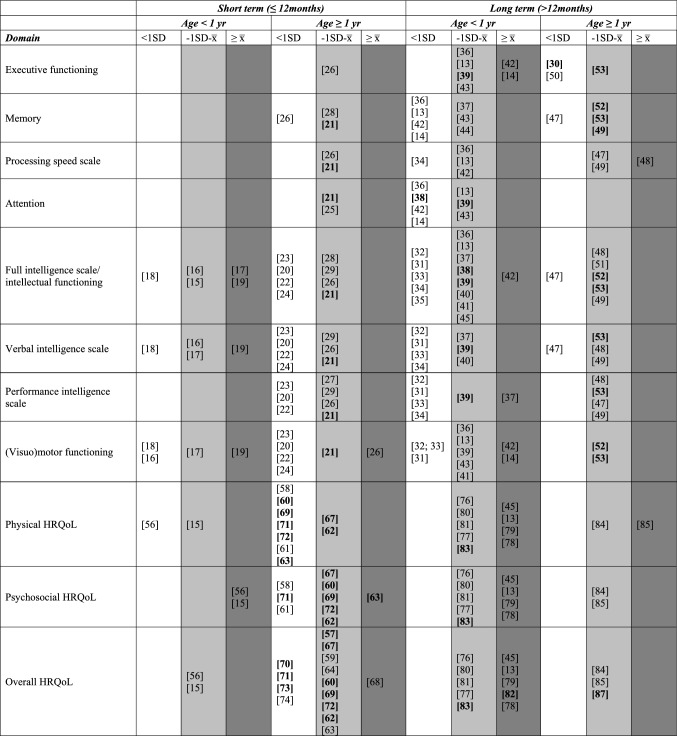
Visual distribution of results of studies included in the systematic review on neurocognitive and HRQoL outcomes

Studies that reported data on differences between PICU patients and healthy control children/normative data are presented in the table. Studies are divided in three groups based on the mean results reported in the studies: *white* representing < 1SD below the average (x̅) of healthy children/norm data, *light gray* representing between 1SD below and average of healthy children/norm data, and *dark gray* comparable or higher scores than average of healthy children/norm data

For studies reporting percentages, the division was made based on the normal distribution in the general population (Online Resource 1b) with 34% scoring between average and 1SD, and 15,7% scoring more than 1SD below healthy children/norm data. When the results of the study reported a percentage that was higher than indicated in that category for healthy children, it was categorized as worse. For example, when 40% of the patients had scores between average and 1SD below average, this was marked as white as it is more than the expected 34% in the light gray column. Study numbers expressed in bold are studies with a large sample size including *n* = 100 patients or more

### Study quality

The methodological quality of the studies was assessed with appropriate scales [12]: the Cochrane Collaboration’s Tool (CCT) for RCTs, the Newcastle–Ottawa Scale (NOS) for cohort studies, and the Agency for Healthcare Research and Quality (AHRQ) methodology checklist for cross-sectional studies [12]. The Cochrane Collaboration’s tool consists of seven components that can be scored with ‘low risk,’ ‘high risk,’ or ‘unclear risk.’ For the current study, for each item judged as ‘high risk,’ one point was assigned, and for ‘low risk’ and ‘unclear risk’ zero points were assigned, which results in a maximum total score of seven points. The NOS consists of eight components and each component has a score of zero or one, with one component a score of zero, one, or two, and a maximum of nine points to achieve. The ARHQ counts 11 items scoring zero or one, which results in a maximum total score of 11 points.

## Results

### Study selection

**Fig. 1:**
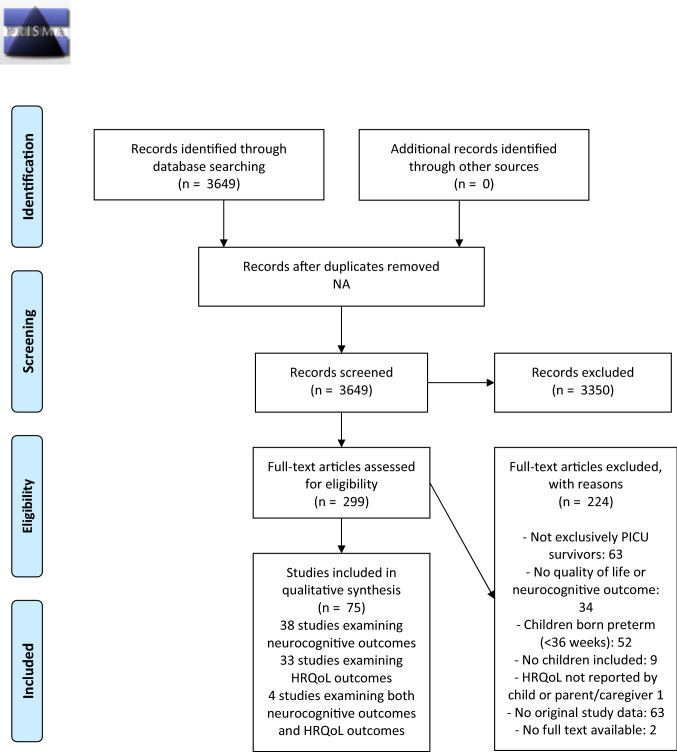
Flowchart of studies included in the systematic review according to the PRISMA statement

A total of 3649 studies were identified. After reviewing title and abstract, 3350 studies were excluded. After full-text screening of the remaining 299 articles for eligibility, a total of 75 articles (38 on neurocognitive functioning, 33 on HRQoL, and 4 on both outcomes) met the inclusion criteria and were included in the qualitative data reviewing. Thirty-eight studies investigated neurocognitive functioning (*Fig. **1*), with sample sizes ranging between 17 and 786, and 32 with less than 100 study patients (Supplemental Table 1). Thirty-three studies examined HRQoL (*Fig. **1*), with sample sizes ranging between 6 and 1109, and 20 studies with less than 100 study patients (Supplemental Table 2). Four studies with sample sizes ranging between 25 and 43 investigated both neurocognitive functioning and HRQoL [13, 14]. Of the 75 articles included, 50 were prospective cohort studies, 6 retrospective cohort studies, 2 cross-sectional studies, and 17 RCTs (Supplemental Tables 1 and 2). Of these RCTs, ten studies reported effects of interventions during PICU admission and seven studies reported effects of interventions during follow-up. Two RCTs investigated psychosocial interventions, 15 RCTs examined the effect of disease management strategies.

### Risk of bias assessment

The average risk of bias score of the 14 RCTs was 3.2 out of seven, with a range of zero to four. Most studies had a low risk of bias regarding the random sequence of the allocation to intervention groups. Almost all studies had problems with blinding participants to intervention groups. The average risk of bias score of the 50 cohort studies was 5.6 out of nine, with a range of three to eight. Selection bias was common due to inclusion criteria such as age restrictions. Furthermore, studies frequently had no adequate follow-up or had no description of the patients lost to follow-up. The risk of bias score of the two cross-sectional studies included in this systematic review were 4 and 6 out of 11 (Supplemental tables 1a, 1b, 2a, and 2b for individual scores of the studies).

### Neurocognitive functioning after PICU admission

#### Short-term follow-up (≤ 12 months) after PICU admission

In patients who were infants (< 12 months) at time of PICU admission, 5 studies were done with a short-term follow-up (Supplemental Table 1a) [15–19]. Three out of these 5 studies encompassed children who underwent surgery for congenital cardiac anomalies. They found that children had general intelligence scores within the normal range (standard scores between 85 and 115, Table 1) [16, 17, 19]. Longer length of PICU stay was correlated with lower general intelligence [16, 17].

In patients one year or older at PICU admission, 11 studies were done (Supplemental Table 1a). Overall, studies reported lower scores compared to normative data regarding intelligence, processing speed, executive functioning, memory, attention, and (visuo)motor functioning (Table 1) [20–30]. In children with traumatic brain injury, 3 studies found lower scores than normative data regarding general intelligence [25–27], processing speed [26], executive functioning [26], working memory [26], and attention [25]. Factors associated with lower scores on attention were younger age at PICU admission, baseline levels of inattention, and higher serum levels of the biomarker neuron-specific enolase [25]. In children who underwent liver transplantation, longer length of PICU stay, longer follow-up time, and higher age were associated with worse executive functioning [30]. In an overlapping sample of children who were comatose after return of spontaneous circulation, five RCTs investigated normothermia versus hypothermia and reported that 33% to 90% of children had general intelligence scores within 1SD below the norm. The effect of the RCT on general intelligence outcomes was not reported [20–24].

#### Long-term follow-up (> 12 months) after PICU admission

In patients who were infants (< 12 months) at the time of PICU admission, 18 long-term studies were done (Supplemental Table 1b). Overall, these studies reported lower scores than normative data on all neurocognitive domains (intelligence, processing speed, executive functioning, memory, attention, and visuo(motor) functioning, Table 1) [13, 14, 31–45]. Patients treated with extracorporeal life support (ECLS) and extracorporeal membrane oxygenation (ECMO) were investigated in 10 of the 18 studies [13, 14, 18, 32, 36, 38, 40, 42–44]. One research group conducted seven studies in an overlapping sample of patients treated with ECMO [13, 14, 36, 38, 42, 44, 46]. Most of these studies reported lower scores than normative data for general intelligence, memory, and attention. These scores were within the normal range, except for attention at eight years follow-up [38], and memory at 18 years follow-up [13], with scores more than 1SD below the norm. In ECLS survivors, factors associated with lower general intelligence scores were lower weight [32], lower SES [32], more cardiac operations and catheterizations [40], and older age at first cannulation [40]. Intelligence was stable over time in patients treated with ECMO, assessed at the age of two, five, and eight years [38]. A study in patients who underwent surgery for congenital heart disease (at 4 years and 7 years of follow-up) with a relatively large sample size (*N* = 107) found that the differences in general intelligence scores between patients and healthy controls decreased over time [39]. The two studies including children with traumatic brain injury at young age found long-term consequences for cognitive and language development [34, 45] that worsen over time [45].

In patients who were one year or older at PICU admission, 8 studies were done (Supplemental Table 1b). Reasons for admission were traumatic brain injury [47], liver transplantation [48, 49], acute neurological conditions [50], postoperative delirium [51], or critical illness in general [52–54]. Overall, PICU survivors obtained lower scores on all neurocognitive domains compared with normative data (Table 1). In a large sample of critically ill children (*N* = 786), two follow-up studies of an RCT concerning the start of supplemental parenteral nutrition (PN) on PICU admission (*early* from day one or *late* after one week) found that late PN was not harmful with regard to neurocognitive outcomes 2 and 4 years later, and led to better scores for visuomotor integration [53], and parent-reported executive functioning [53], internalizing problems [54], and externalizing problems [53, 54]. Overall, patients scored lower than matched healthy control children on neurocognitive outcomes [53, 54]. Factors associated with lower neurocognitive scores were longer length of PICU stay [51], more circulating phthalates [52], use of benzodiazepines [53, 54], and corticosteroids [53] during PICU admission, as well as higher premorbid developmental risk [51]. Use of alpha2 agonists during PICU admission [53, 54] was associated with better scores.

### HRQoL after PICU admission

#### Short-term follow-up (≤ 12 months) after PICU admission

In patients who were infants (< 12 months) at the time of PICU admission, 3 studies were done (Supplemental Table 2a) [15, 55, 56]: all 3 studies found lower HRQoL scores compared with norm data on the Infant Toddler Quality of life Questionnaire (ITQoL), see Table 1. A daily sedation interruption intervention in children who required mechanical ventilation did not influence HRQoL on the short-term compared with protocolized sedation [56]. In children who survived a cardiac arrest, parenteral burden scale scores on the child’s HRQoL questionnaire were worse than normative data [55]. Although scores remained below the norm, parental burden improved in the first year after PICU admission [55].

In patients who were one year or older at PICU admission, 18 studies were done (Supplemental Table 2a) [57–74]. Eleven of the 18 studies compared patients with normative data or healthy control children and found lower physical HRQoL scores [57–65, 69, 72] and 10 of these 11 studies found overall lower psychosocial HRQoL [57–60, 62–65, 69, 72]. Four studies in the same pediatric sepsis cohort found lower parent-reported physical and psychosocial HRQoL than norm data [69, 70, 73, 74]. Risk factors were neurological complications during hospitalization, dependence on a medical device 1 month post admission [69], and single parent families [70]. Risk factors for HRQoL at 3 months were magnitude/duration of organ dysfunction, duration of hospitalization, and older age [73]. Of the total sample, 35% had deterioration in HRQoL that persisted up to 1 year after admission [74].

Four studies investigated the complete group of critically ill children irrespective of diagnosis [57–60]. One of these studies found that parents’ own mental health was associated with lower HRQoL scores they reported for their child [60]. During the first year after critical illness, physical HRQoL of critically ill children improved more than their psychosocial HRQoL [57]. Factors associated with lower scores for physical and psychosocial domains in the complete group of critically ill children were older age, longer length of PICU stay, and worse disease severity [60]. In children with a brain insult, an RCT revealed that early rehabilitation (consisting of physical therapy, occupational therapy, and speech and language therapy) was not superior to standard care in improving HRQoL [64]. In children with acute respiratory failure, nurse-implemented, goal-directed sedation versus usual care in children with acute respiratory failure had overall no effect on HRQoL outcomes [62]. For severely burned children, a community-based exercise program improved some physical and psychosocial HRQoL scales compared with a hospital based exercise program [66].

#### Long-term follow-up (> 12 months) after PICU admission

In patients who were infants (< 12 months) at the time of PICU admission, 12 studies were done (Supplemental Table 2b) [13, 14, 45, 75–83]. Five [75–77, 80, 81] studies found overall lower physical and psychosocial HRQoL scores compared with normative data, see Table 1. The admission reasons vary between studies: after ECMO treatment [76, 77, 81], for cardiac arrest [81], and for perinatal asphyxia [75]. Children who were treated with ECMO obtained lower physical and psychosocial HRQoL scores compared with normative data; 43% of the neonates treated with ECMO had scores below 1SD of normative data at 5 years of follow-up [81], 90% of the neonates treated with ECMO had no disabilities 20 years later [76], and patients treated with ECMO younger than 13 years scored lower than normative data, whereas survivors 13 years or older did not [77]. One study in children with abusive head trauma overall fond better parent-reported HRQoL [45]. A study in a heterogeneous group of critically ill children found that 93% had an overall good HRQoL [82]. Factors associated with lower outcomes in children aged younger than 12 months were need of mechanical ventilation [82] and neurological complications (particularly intracranial ischemia or hemorrhage) [81]. Three studies investigated the discrepancy between child reports and parent reports [78–80] of which 2 studies reported better self-reported than parent-reported HRQoL in children with a cardiac arrest and in those needing mechanical circulatory support [78, 80].

In patients who were one year or older at PICU admission, 5 studies were done (Supplemental Table 2b) [83–87]. One study in critically ill children in general with a large sample size (*N* = 1109) [87] reported that approximately 10% of the children scores more than 2SD below the norm for physical and psychosocial HRQoL, associated with an elective admission and a neurological diagnosis [87]. Another large study (*n* = 786) found overall lower HRQoL in a heterogeneous group of critically ill children compared with n = 406 healthy matched control children. Additionally, in younger children worse growth and development scores and older children worse role functioning and mental health scores were found [83]. The other (smaller) studies (in children with cerebral palsy after spinal arthrodesis, patients treated with ECMO, and children with traumatic brain injury) also found lower physical HRQoL and psychosocial HRQoL [84–86].

## Discussion

This systematic review found overall lower scores for neurocognitive functioning and HRQoL in children admitted to the PICU compared to healthy control children or normative data from the general population. However, many studies reported mean or median scores for neurocognitive functioning within the normal range. Scores that fall within the normal range are scores within 1SD above or below the norm mean. With regard to HRQoL, most studies found that more than half of the children had an average HRQoL after PICU admission.

Studies that investigated the short-term follow-up of patients who were infants at PICU admission are limited to 5 published studies. The reason for this lack of studies in infants is understandable for neurocognitive functioning due to the difficulties in conducting neurocognitive assessment in infants and young children. These difficulties are a result of incomplete brain development of most domains of neurocognitive functioning below the age of five years old. Therefore, general intellectual functioning can be assessed in these young children, but for example executive functioning can not. However, for HRQoL no age restrictions exist, and parent-reported measurements are also validated for young children [88]. To have a more complete view of the first year after critical illness in infants, more studies should focus on development and HRQoL. In both infants and older children, studies focusing on HRQoL reported lower scores than the norm in the short term, but in the long term, a substantial part of the studies reported HRQoL scores comparable or even better than the norm. This could possibly be explained by the biopsychosocial model for recovery as published by Atkins et al. [3]. This model explains that after PICU discharge recovery takes place in three domains: physical functioning, emotional functioning, and social functioning. After a longer period of time, recovery in these domains leads to a response shift, that includes adjustments to shift from experiencing a traumatic event to returning to ‘normal’ life. Thus, although these children might still be impaired in domains of post-intensive care syndrome (PICS-p) [89], such as neurocognitive functioning, how they cope with their impairments (or how parents appraise these impairments in their child) is adapted and is not evaluated as impaired any longer [3]. On the other hand, it could also be possible that impairments disappear over time and do not affect daily life any longer.

### Interventions on the PICU and follow-up

A number of factors were reported to predict worse neurocognitive and HRQoL outcomes after PICU admission. These predictors include pre-existing factors (such as lower weight, lower SES), but also factors related to the management at the PICU (such as use of benzodiazepines and corticosteroids), of which the latter are modifiable. In infants at time of PICU admission, longer length of PICU stay was associated with worse general intelligence scores in the short term (≤ 12 months after PICU admission) [16, 17]. In the long term (> 12 months after PICU admission), worse scores in infants were predicted by lower weight [32], lower SES [32], and older age [40] predicted worse general intelligence scores. In children aged 12 months or older at the time of PICU admission factors that were associated with worse neurocognitive scores were higher baseline levels of inattention [25], higher serum levels of the biomarker neuron-specific enolase [25], longer length of PICU stay [30], and longer follow-up time [30] on the short term. In one study younger age at PICU admission was reported to predict worse scores for attention [25], but in another study higher age predicted worse scores for executive functioning [30]. This difference could be related to the difference in domains or to the difference in reason for admission, respectively, traumatic brain injury [25] and liver transplantation [30]. In the long term, longer length of PICU stay [51], more circulating phthalates [52] and use of benzodiazepines and corticosteroids during PICU admission [53], and higher premorbid developmental risk [51] were associated with worse neurocognitive scores. For HRQoL, factors associated with lower scores on the long term for infants were the need of mechanical ventilation [82] and neurological complications (intracranial ischemia or hemorrhage) [81]. For children aged 12 months or older, older age, longer length of stay and worse disease severity [60] were associated with lower HRQoL in the short term, and in the long term an elective PICU admission and neurological diagnosis [87]. In conclusion, these predictors are pre-existing factors or factors related to the management at the PICU, which may be modifiable.

Some studies have tried to influence these factors through psychosocial and disease management interventions, both during PICU admission and follow-up, to improve short-term and long-term outcomes related to neurocognitive functioning and HRQoL. Starting late with supplemental parenteral nutrition during the first week of critical illness compared to starting early had a positive effect on some neurocognitive domains [53, 54]. Hypothermia versus normothermia [75] and different sedation procedures did not reveal differences in HRQoL outcomes [56, 62, 63]. Two RCTs tried to improve HRQoL by rehabilitation [64, 66] and found that, after PICU discharge, an exercise program near home was slightly more effective than a program at the hospital [66] and that early protocolized rehabilitation (< 72 h of PICU admission) was not better than usual care [64]. A working memory training (Cogmed) improved working memory only immediately after the training, and the training had no effect on HRQoL outcomes [14]. Although some intervention studies tried to improve outcomes by affecting predictors, there is a lack of research on mitigating adverse PICS-p outcomes in critically ill children using RCTs. One of the under investigated fields is the importance of parents in the recovery of critically ill children after PICU discharge, investigating for example the effect of monitoring and empowering parents on the PICU through shared decision making and the influence on PICS-p outcomes after PICU discharge [90].

### Implications for follow-up

The survival rates at the PICU have increased in the last decades [91],which presents the challenge of limiting morbidity related to PICS-p. Some critically ill children obtained worse neurocognitive functioning and HRQoL outcomes compared with children from the general population. It is important to gain insight in this group of critically ill children and their parents to prevent cognitive problems or psychiatric problems such as anxiety, depression, or posttraumatic stress they may develop after PICU admission of the child [92]. Psychoeducation and parent support (teaching skills and emotional support) during PICU admission and directly after discharge are important [93]. During follow-up, all children and families should be monitored neurocognitive and psychosocially to find those at risk for impaired development and developing morbidities. Since some children and parents have delayed reactions with regard to psychosocial symptoms [94], this monitoring should be done both short term and long term [6]. Furthermore, psychosocial interventions should be offered to families at high risk for psychosocial problems. Although some studies reported predictors for parents at risk, for example parents with higher baseline stress levels, there is also a lack of multi-factorial predictive models for parental and children’s outcomes [93]. Such predictive models might guide (neuro)psychologists, involved in providing follow-up care, in selecting accurate neurocognitive tests, including assessment of attention, memory, and/or executive functioning, and validated HRQoL questionnaires and other psychosocial questionnaires to monitor psychiatric problems. Moreover, these predictive models might guide which psychosocial innervations might be deployed. However, there is also a lack of research into the effectiveness of psychosocial interventions critically ill children and their families with high risk for psychosocial problems.

With regard to the neurocognitive test battery used in the studies, only one research group assessed different domains of neurocognitive functioning (as part of a structured longitudinal clinical follow-up program) at different time points until the age of 18 years which was in a group of patients treated with ECMO [13, 14, 36, 38, 42, 43]. Overall, they found impairments in general intellectual functioning, attention, memory, and executive functioning. Interestingly, the other studies included in this review that investigated neurocognitive functioning assessed general intellectual functioning only, or exclusively investigated a certain domain of neurocognitive functioning (Table 1). General intellectual functioning is usually called intelligence and includes abilities (such as logical reasoning, problem-solving, learning, and verbal skills) that allows a person to understand the reality and to interact with it [95]. Although general intellectual functioning is predictive of school functioning, it is important to understand which, more specific, neurocognitive domain has been impaired. In neuropsychology, the model of hierarchy of neurocognitive domains has often been used to map developmental problems of the child. In this neurocognitive hierarchy, lower functions, such as sensation and attention, affect the outcomes of the higher neurocognitive domain memory. Impairments in memory functioning affects, in turn, the development of executive functioning [96]. When impairments exist in the lower domains of the hierarchy, patients will experience more consequences in daily life as it will affect the higher neurocognitive domains [96]. Therefore, it is important to assess all layers of the hierarchy to figure out where the problem exists.

HRQoL is usually assessed by the parent or caregiver, as young children are not able to report on their own HRQoL. Studies that also included children who were old enough to self-report, found that parents’ proxy-reports were worse than self-reports [78, 80]. Furthermore, the mental HRQoL of the parent itself was a predictor of HRQoL scores they reported for their child, which is a result of shared variance of the informant [60]. The value of proxy-reports is often debated. A systematic review examining the similarities and differences between reports of children and their parents concluded that it is not the question who of the informants is right, but that the discrepancies in outcomes between informants are rather a reflection of differences in their perspectives which gives valued information about the parent and the child [97]. Therefore, a multi-informant approach is recommended (both child and parent PROMs) in daily clinical practice.

### Strengths and limitations

A strength of the current study is that the literature search was conducted using rigorous methodology with the support of a librarian. Furthermore, it describes the legacy of a PICU admission on the crucial domains of daily life, namely cognitive, physical, emotional, and social health, described as PICS-p [98]. This systematic review investigated all these domains, including HRQoL which is the subjective evaluation of physical, emotional, and social health. Although one reviewer discussed all the full texts with another reviewer, a limitation is that not both reviewers read all the full texts. Furthermore, a second weakness is the large heterogeneity across studies with regard to study design, domains assessed, instruments used, analysis methods, and reporting results. Furthermore, most studies did not report standard scores, which makes it hard to compare results between studies. The majority of studies focused on specific diseases or diagnoses which makes it impossible to draw firm conclusions. Previous reviews excluded studies focusing on electively admitted children or on specific diagnosis [5, 89, 99], or excluded neonates admitted to the PICU [100]. Studies included in this systematic review are representative for the complete group of PICU survivors as no patient populations were excluded and most common reasons for a PICU admission were covered, particularly respiratory disease, cardiac disease, and neurologic disorders [101]. Lastly, the quality of studies varied with a number of studies having a high risk of bias, urging for high-quality future research.

## Conclusions

This study pointed out that the population of PICU survivors, overall, experiences worse neurocognitive functioning and HRQoL compared to children from the general population, both in the short term and long term. These morbidities that children experience after PICU discharge are previously described as PICS-p which includes physical, cognitive, emotional, and social problems. A number of RCTs have tried to influence PICS-p during or after PICU admission and showed varying results regarding long-term follow-up. More research is needed into interventions, tested in RCTs, aiming on preventing or improving impairments in neurocognitive functioning and HRQoL. Moreover, more research is needed into predicting which critically ill children are at risk for worse PICS-p outcomes and might benefit from these interventions.

## Supplementary Information

Below is the link to the electronic supplementary material.

Supplementary material 1 (DOCX 109 kb)

## Data Availability

Not applicable.
