# Correction to: Neurocognitive functioning and health‑related quality of life of children after pediatric intensive care admission: a systematic review

**DOI:** 10.1007/s11136-022-03144-9

**Published:** 2022-05-09

**Authors:** José A. Hordijk, Sascha C. Verbruggen, Corinne M. Buysse, Elisabeth M. Utens, Koen F. Joosten, Karolijn Dulfer

**Affiliations:** 1 Intensive Care, Department of Pediatrics and Pediatric Surgery, Erasmus MC - Sophia Children’s Hospital, Dr. Molewaterplein 60, 3015 GJ Rotterdam, The Netherlands; 2 Research Institute of Child Development and Education, University of Amsterdam, Nieuwe Achtergracht 127, 1018 WS Amsterdam, The Netherlands; 3 Academic Center for Child Psychiatry the Bascule/Department of Child and Adolescent Psychiatry, Academic Medical Center, Rijksstraatweg 145, 1115 AP Amsterdam, The Netherlands; 4 Department of Child and Adolescent Psychiatry/Psychology, Erasmus MC - Sophia Children’s Hospital, Wytemaweg 8, 3015 CN Rotterdam, The Netherlands

## Correction to: Quality of Life Research 10.1007/s11136-022-03124-z

In the original publication, the incorrect version of Table 1 was inadvertently published. The correct version of Table 1 is provided below.

**Table 1:**
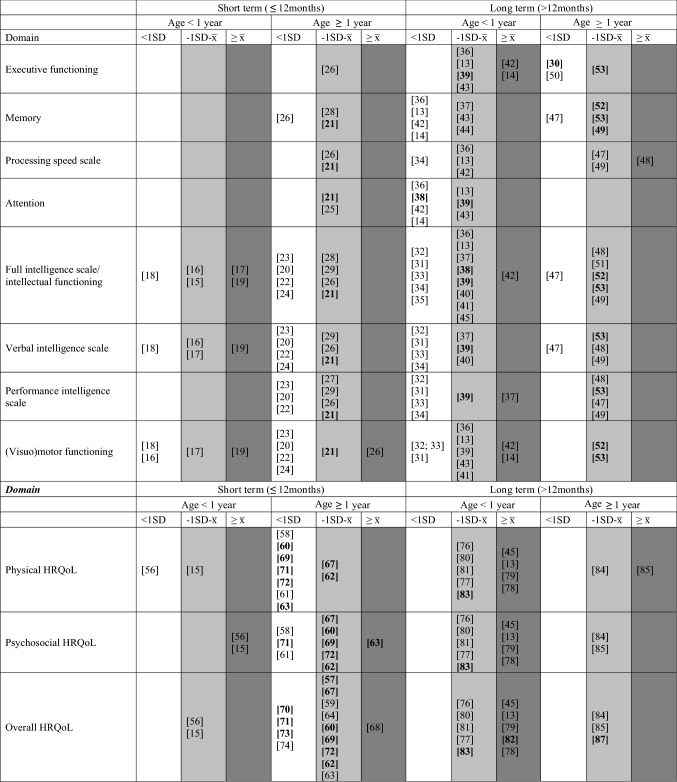
Visual distribution of results of studies included in the systematic review on neurocognitive and HRQoL outcomes

Studies that reported data on differences between PICU patients and healthy control children/normative data are presented in the table. Studies are divided in three groups based on the mean results reported in the studies: *white* representing < 1SD below the average (x̅) of healthy children/norm data, *light gray* representing between 1SD below and average of healthy children/norm data, and *dark gray* comparable or higher scores than average of healthy children/norm data

For studies reporting percentages, the division was made based on the normal distribution in the general population (Online Resource 1b) with 34% scoring between average and 1SD, and 15,7% scoring more than 1SD below healthy children/norm data. When the results of the study reported a percentage that was higher than indicated in that category for healthy children, it was categorized as worse. For example, when 40% of the patients had scores between average and 1SD below average, this was marked as white as it is more than the expected 34% in the light gray column. Study numbers expressed in bold are studies with a large sample size including *n* = 100 patients or more

The original article has been corrected.

