# Supplementary material for: Neurocognitive functioning and health-related quality of life of children after pediatric intensive care admission: a systematic review
Source: Qual Life Res. 2022 Mar 31;31(9):2601–14. doi: 10.1007/s11136-022-03124-z (PMC9356943; doi:10.1007/s11136-022-03124-z)
Supplement: Supplementary file 1 — Supplementary material 1 (DOCX 109 kb) [file 11136_2022_3124_MOESM1_ESM.docx]

**Supplementary appendix**

**Neurocognitive functioning and health-related quality of life of children after pediatric intensive care admission: a systematic review**

José A. Hordijk^1^, MSc, Sascha C. Verbruggen^1^, MD, PhD, Corinne M. Buysse^1^, MD, PhD, Elisabeth M. Utens^2,3,4^, PhD, Koen F. Joosten^1^, MD, PhD, Karolijn Dulfer^1^, PhD

**Affiliations**

^1^ Intensive Care, Department of Pediatrics and Pediatric Surgery, Erasmus MC - Sophia Children’s Hospital, Dr. Molewaterplein 60, 3015 GJ, Rotterdam, The Netherlands

^2^ Research Institute of Child Development and Education, University of Amsterdam, Nieuwe Achtergracht 127, 1018 WS, Amsterdam, The Netherlands

^3^ Academic Center for Child psychiatry the Bascule/Department of Child and Adolescent Psychiatry, Academic Medical Center, Rijksstraatweg 145, 1115 AP, Amsterdam, The Netherlands
^4^ Department of Child and Adolescent Psychiatry/Psychology, Erasmus MC - Sophia Children’s Hospital, Wytemaweg 8, 3015 CN, Rotterdam, The Netherlands

**Supplementary material**

**Pg 3 Online Resource 1a. Search strategy**

**Pg 7 Online Resource 1b. Explanation colors in table S3 based on the normal distribution of**

**scores in the general population**

**Pg 8 Supplemental table 1a. Included studies on short-term (≤12 months) neurocognitive outcomes after PICU**

**Pg 15 Supplemental table 1b. Included studies on long-term (>12 months) neurocognitive outcomes after PICU**

**Pg 28 Supplemental table 2a. Included studies on short-term (≤12 months) health-related quality of life outcomes after PICU**

**Pg 38 Supplemental table 2b. Included studies on long-term (>12 months) health-related quality of life outcomes after PICU**

**P 46 References**

**Online Resource 1a. Search strategy**

**Embase.com**

('intensive care'/de OR 'intensive care unit'/de OR 'critical illness'/de OR 'respiratory care'/de OR 'pediatric intensive care unit'/de OR 'central venous catheter'/exp OR 'central venous catheterization'/de OR 'Neisseria meningitidis'/de OR 'meningococcosis'/exp OR 'shock'/de OR 'septic shock'/de OR 'toxic shock syndrome'/de OR 'endotracheal intubation'/exp OR 'artificial ventilation'/de OR 'respiratory failure'/exp OR 'intubation'/exp OR (((intensive OR critical*) NEAR/3 (care OR illness* OR ill)) OR 'respiratory care' OR (central* NEAR/3 venous NEAR/3 catheter*) OR 'Neisseria meningitidis' OR meningococcos* OR (meningococ* NEAR/3 infect*) OR (septic* NEAR/3 shock) OR ((endotrach* OR intratrach*) NEAR/3 intubat*) OR (artificial NEAR/3 (ventilat* OR repirator*)) OR ((circulat* OR respirator*) NEAR/3 (insufficie* OR failure*)) OR intubat*):ab,ti) AND (child/exp OR adolescent/exp OR adolescence/exp OR 'child behavior'/de OR 'child parent relation'/de OR pediatrics/exp OR childhood/exp OR 'child nutrition'/de OR 'infant nutrition'/exp OR 'child welfare'/de OR 'child abuse'/de OR 'child advocacy'/de OR 'child development'/de OR 'child growth'/de OR 'child health'/de OR 'child health care'/exp OR 'child care'/exp OR 'childhood disease'/exp OR 'child death'/de OR 'child psychiatry'/de OR 'child psychology'/de OR 'pediatric ward'/de OR 'pediatric hospital'/de OR 'pediatric anesthesia'/de OR 'pediatric intensive care unit'/de OR (adolescen* OR preadolescen* OR infan* OR child* OR kid OR kids OR toddler* OR teen* OR boy* OR girl* OR minors OR underag* OR (under NEXT/1 (age* OR aging)) OR juvenil* OR youth* OR kindergar* OR puber* OR pubescen* OR prepubescen* OR prepubert* OR pediatric* OR paediatric* OR schoolchild* OR 'school child*' OR preschool* OR highschool*):ab,ti) AND ('cognitive defect'/de OR 'mild cognitive impairment'/de OR 'cognition'/de OR 'memory disorder'/de OR 'memory'/de OR 'impulsiveness'/exp OR 'impulse control disorder'/exp OR 'intelligence quotient'/de OR 'quality of life'/exp OR 'problem solving'/de OR 'neuropsychological test'/exp OR 'speech and language'/de OR 'language'/de OR 'speech'/exp OR 'speech and language assessment'/exp OR 'visuomotor coordination'/de OR (cogniti* OR neurocogniti* OR memory OR amnesia OR paramnesia OR adhd OR impulsiv* OR (impulse NEAR/3 control) OR ((Neuropsychologic* OR Executive* OR cogniti* OR Neurobehav* OR attention* ) NEAR/3 (outcome* OR disorder* OR function* OR dysfunction* OR morbidit* OR impair* OR perform* OR develop* OR status OR deficit* OR problem* OR limitation*)) OR 'intelligence quotient' OR iq OR (quality NEAR/3 life) OR 'problem solving' OR speech OR language OR visuomotor* OR (visual NEAR/3 spatial ) OR (information NEAR/3 processing) OR ((constructive OR decision* OR verbal) NEAR/3 skill*) OR inhibition):ab,ti) NOT ([Conference Abstract]/lim OR [Letter]/lim OR [Note]/lim OR [Editorial]/lim) AND [english]/lim

**Medline Ovid**

(Critical Care/ OR Intensive Care Units/ OR Intensive Care Units, Pediatric/ OR Respiratory Care Units/ OR Critical Illness/ OR Central Venous Catheters/ OR Catheterization, Central Venous/ OR Neisseria meningitidis/ OR Meningococcal Infections/ OR shock/ OR Shock, Septic/ OR Intubation, Intratracheal/ OR exp Respiration, Artificial/ OR Ventilators, Mechanical/ OR Respiratory Insufficiency/ OR exp intubation/ OR (((intensive OR critical*) ADJ3 (care OR illness* OR ill)) OR respiratory care OR (central* ADJ3 venous ADJ3 catheter*) OR Neisseria meningitidis OR meningococcos* OR (meningococ* ADJ3 infect*) OR (septic* ADJ3 shock) OR ((endotrach* OR intratrach*) ADJ3 intubat*) OR (artificial ADJ3 (ventilat* OR repirator*)) OR ((circulat* OR respirator*) ADJ3 (insufficie* OR failure*)) OR intubat*).ab,ti.) AND (exp Child/ OR Infant/ OR exp Adolescent/ OR exp "Child Behavior"/ OR exp "Parent Child Relations"/ OR exp "Pediatrics"/ OR "Child Nutrition Sciences"/ OR "Infant nutritional physiological phenomena"/ OR exp "Child Welfare"/ OR "Child Development"/ OR exp "Child Health Services"/ OR exp "Child Care"/ OR "Child Rearing"/ OR exp "Child development Disorders, Pervasive"/ OR "Child Psychiatry"/ OR "Child Psychology"/ OR "Hospitals, Pediatric"/ OR exp "Intensive Care Units, Pediatric"/ OR (adolescen* OR preadolescen* OR infan* OR child* OR kid OR kids OR toddler* OR teen* OR boy* OR girl* OR minors OR underag* OR (under ADJ (age* OR aging)) OR juvenil* OR youth* OR kindergar* OR puber* OR pubescen* OR prepubescen* OR prepubert* OR pediatric* OR paediatric* OR schoolchild* OR school child* OR preschool* OR highschool*).ab,ti.) AND (Cognitive Dysfunction/ OR cognition/ OR Memory Disorders/ OR memory/ OR Impulsive Behavior/ OR Disruptive, Impulse Control, and Conduct Disorders/ OR intelligence/ OR Quality of Life/ OR Problem Solving/ OR exp neuropsychological test/ OR Speech-Language Pathology/ OR language/ OR speech/ OR (cogniti* OR neurocogniti* OR memory OR amnesia OR paramnesia OR adhd OR impulsiv* OR (impulse ADJ3 control) OR ((Neuropsychologic* OR Executive* OR cogniti* OR Neurobehav* OR attention* ) ADJ3 (outcome* OR disorder* OR function* OR dysfunction* OR morbidit* OR impair* OR perform* OR develop* OR status OR deficit* OR problem* OR limitation*)) OR intelligence quotient OR iq OR (quality ADJ3 life) OR problem solving OR speech OR language OR visuomotor* OR (visual ADJ3 spatial ) OR (information ADJ3 processing) OR ((constructive OR decision* OR verbal) ADJ3 skill*) OR inhibition).ab,ti.) NOT (letter OR news OR comment OR editorial OR congresses OR abstracts).pt. AND english.la.

**Cochrane CENTRAL**

((((intensive OR critical*) NEAR/3 (care OR illness* OR ill)) OR 'respiratory care' OR (central* NEAR/3 venous NEAR/3 catheter*) OR 'Neisseria meningitidis' OR meningococcos* OR (meningococ* NEAR/3 infect*) OR (septic* NEAR/3 shock) OR ((endotrach* OR intratrach*) NEAR/3 intubat*) OR (artificial NEAR/3 (ventilat* OR repirator*)) OR ((circulat* OR respirator*) NEAR/3 (insufficie* OR failure*)) OR intubat*):ab,ti) AND ((adolescen* OR preadolescen* OR infan* OR child* OR kid OR kids OR toddler* OR teen* OR boy* OR girl* OR minors OR underag* OR (under NEXT/1 (age* OR aging)) OR juvenil* OR youth* OR kindergar* OR puber* OR pubescen* OR prepubescen* OR prepubert* OR pediatric* OR paediatric* OR schoolchild* OR 'school child*' OR preschool* OR highschool*):ab,ti) AND ((cogniti* OR neurocogniti* OR memory OR amnesia OR paramnesia OR adhd OR impulsiv* OR (impulse NEAR/3 control) OR ((Neuropsychologic* OR Executive* OR cogniti* OR Neurobehav* OR attention* ) NEAR/3 (outcome* OR disorder* OR function* OR dysfunction* OR morbidit* OR impair* OR perform* OR develop* OR status OR deficit* OR problem* OR limitation*)) OR 'intelligence quotient' OR iq OR (quality NEAR/3 life) OR 'problem solving' OR speech OR language OR visuomotor* OR (visual NEAR/3 spatial ) OR (information NEAR/3 processing) OR ((constructive OR decision* OR verbal) NEAR/3 skill*) OR inhibition):ab,ti)

**Web of science**

TS=(((((intensive OR critical*) NEAR/2 (care OR illness* OR ill)) OR "respiratory care" OR (central* NEAR/2 venous NEAR/2 catheter*) OR "Neisseria meningitidis" OR meningococcos* OR (meningococ* NEAR/2 infect*) OR (septic* NEAR/2 shock) OR ((endotrach* OR intratrach*) NEAR/2 intubat*) OR (artificial NEAR/2 (ventilat* OR repirator*)) OR ((circulat* OR respirator*) NEAR/2 (insufficie* OR failure*)) OR intubat*)) AND ((adolescen* OR preadolescen* OR infan* OR child* OR kid OR kids OR toddler* OR teen* OR boy* OR girl* OR minors OR underag* OR (under NEAR/1 (age* OR aging)) OR juvenil* OR youth* OR kindergar* OR puber* OR pubescen* OR prepubescen* OR prepubert* OR pediatric* OR paediatric* OR schoolchild* OR "school child*" OR preschool* OR highschool*)) AND ((cogniti* OR neurocogniti* OR memory OR amnesia OR paramnesia OR adhd OR impulsiv* OR (impulse NEAR/2 control) OR ((Neuropsychologic* OR Executive* OR cogniti* OR Neurobehav* OR attention* ) NEAR/2 (outcome* OR disorder* OR function* OR dysfunction* OR morbidit* OR impair* OR perform* OR develop* OR status OR deficit* OR problem* OR limitation*)) OR "intelligence quotient" OR iq OR (quality NEAR/2 life) OR "problem solving" OR speech OR language OR visuomotor* OR (visual NEAR/2 spatial ) OR (information NEAR/2 processing) OR ((constructive OR decision* OR verbal) NEAR/2 skill*) OR inhibition)) ) AND DT=(article) AND LA=(english)

**Google scholar**

"intensive|critical care|illness"|"respiratory care" adolescents|infants|children cognition|memory|adhd|impulsiveness|Neuropsychological|"Executive outcome|disorders| function|dysfunction"

**Online Resource 1b. Explanation colors in table 1 based on the normal distribution of scores in the general population**


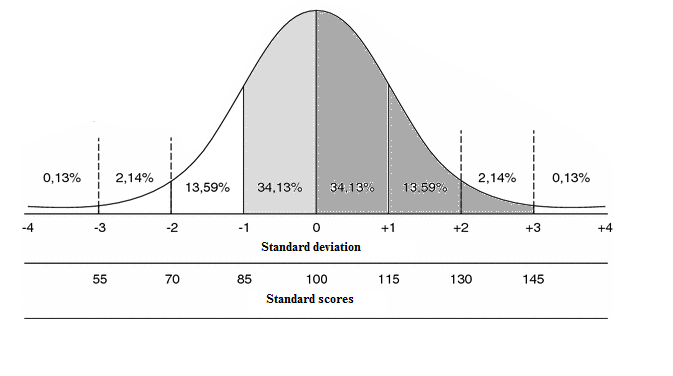


The dark grey columns in Table 1 represent studies with comparable or higher average scores than healthy children/norm data, the light grey column represents study average scores between comparable and 1SD below healthy children/norm data, and the white column represents study average scores more than 1SD below healthy children/norm data. In case of percentages, these percentages were compared with those expected in the general population based on the normal distribution with 34% scoring between average and 1SD (light grey column), and 15,7% scoring more than 1SD below healthy children/norm data (white column)

**Supplemental table 1a. Included studies on short-term (≤12 months) neurocognitive outcomes after PICU**

| *Authors* | *Study population* | *Age at PICU admission* | *Sample size* | *Study design (quality score)* | *Follow-up* | *Measurement* | *Main results* |
| --- | --- | --- | --- | --- | --- | --- | --- |
| Studies including children ≤12 months at time of PICU admission | | | | | | | |
| Bembea et al. 2020 [15] | Children who underwent ECMO for more than 24 hours for any indication | Median 35 [2 days – 20 months] days | 25 | Prospective cohort study (NOS 8/9) | 6 & 12 months | Mullen Scales of Early Learning (<6 years), WISC-IV (≥6 years, intelligence) | 29 patients <6 years with median score 85 [IQR 72-96], 3 patients ≥6 years with scores on FIQ: 90, 89 and 107 |
| Khalid et al. 2019 [16] | Children with single ventricle who underwent a neonatal hybrid procedure | Gestational age median 38.3 [IQR 37.5-39.0] weeks | 24 | Retrospective cohort study (NOS 6/9) | 12 months | Bayley Scales of Infant Development III (Bayley-III, intellectual functioning) | Cognitive score 95, language score 94, motor score 82; elevated lactate, longer mechanical ventilation, length of ICU & hospital stay associated with lower scores |
| Meuwly et al. 2019 [17] | Children with severe congenital heart disease who underwent cardiopulmonary bypass surgery | Median 12 [IQR 9-16] days | 77 | Prospective cohort study (NOS 8/9) | 12 months | Bayley-III (intellectual functioning) | Cognition 105; language 93; motor 93; length of ICU stay negatively associated with cognitive & motor score; postoperative total brain volume & cortical, frontal, temporal, white matter and cerebellar volume positively associated with neurodevelopmental outcome |
| Purkayastha et al. 2018 [18] | Asphyxiated neonates | Gestational age range 37-41 weeks | 34 | Prospective cohort study (NOS 5/9) | 10-14 months | Bayley-III (intellectual functioning) | 24% developmental delay in cognition (Bayley score <85), 41% score of <85 in motor domain, 26% score of <85 in language domain; motor domain was most affected; concerning all 3 domains 59% normal |
| Wei et al. 2019 [19] | Infants with congenital heart disease who underwent cardiac surgery | Mean 12.3 (SD 5.8) months | 106 | Retrospective cohort study (NOS 4/9) | Preoperative, 3 days & 3 months | Bayley-III (intellectual functioning) | 50 infants underwent anesthesia using dexmedetomidine and fentanyl combined with isoflurane & 56 infants in the control group received midazolam and fentanyl combined with isoflurane; scores preoperative, 3 days & 3 months intervention group: cognitive score 104, 98, 104; language score 105, 98, 105; action 103, 97, 103; emotional 102, 91, 102; behavior 101, 92, 102; control group scores; cognitive score 103 97 103; language score 104 96 104; action score 102 95 102; emotional score 102 91 98; behavior score 100 91 100; overall no differences on Bayley for two groups |
| Studies including children >12 months at time of PICU admission | | | | | | | |
| Als et al. 2015 [28] | Children with neuropsychological deficits 3–6 months following meningoencephalitis, sepsis, or other critical illness excluding other primary neurological disorder | Median 6 [IQR 6-13] years | 23 | Prospective cohort study (NOS 4/9) | 3-6 months & 12 months | Wide Range Intelligence Test (5 years old) or Wechsler Abbreviated Scale of Intelligence (>5 years), Children’s Memory Scale (CMS, memory), Cambridge Neuropsychological Test Automated Battery (attention and memory) | No differences in FIQ and visual attention between 3 months follow-up (FIQ = 92) & 12-months follow-up (FIQ = 93); statistically significant improvements in verbal and visual memory tests, CMS immediate index 91 (3 months) and 99 (12 months), delayed index 92 (3 months) and 101 (12 months), recognition 91 (3 months) and 97 (12 months) |
| Christensen et al. 2019 [24] | Comatose children after cardiac arrest and preexisting neurobehavioral impairment (RCT: therapeutic hypothermia 33.0ºC vs. normothermia 36.8ºC) | Mean 4.6 (SD 5.36) years | 28 | RCT (CCT 4/7) | 12 months | Mullen Scales of Early Learning (intellectual functioning) | Scores more than 1SD below the norm: early learning composite 88%, visual perception 88%, fine motor 88%, receptive language 94%, expressive language 95% |
| Dennis et al. 2015 [27] | Children with moderate or severe traumatic brain injury | Slow interhemispheric transfer time (IHTT): mean 13.9 (SD 2.5) years, normal IHTT: mean 14.5 (SD 3.2) years | 32 patients, 31 healthy control children | Prospective cohort study (NOS 7/9) | 1-5 months | Processing Speed Index and Working Memory Index of the WISC-IV or Wechsler Adult Intelligence Scale, 3^rd^ edition (WAIS-III, intelligence), Trials 1–5 from the California Verbal Learning Test–Children’s Version (CVLT-C, memory), second edition, Trial 4 from the Delis-Kaplan Executive Function System (executive functioning) | IHTT slow (performance index = 91) significantly poorer performance index than the IHTT normal group (performance index = 100); whose performance index slightly poorer than healthy control children (performance index = 104) |
| Goldschmidt et al. 2019 [30] | Children who underwent liver transplantation | Age at follow-up: median 10.4 [range 2.1-18.3] years | 155 patients, 296 healthy control children | Prospective cohort study (NOS 5/9) | Median 5.0 (range 0.1-17) months | Children’s Color Trail Test (CCTT) & cognitive Pediatric Quality of Life Inventory (PedsQL) parent and child form (executive functioning) | Subtests of the CCTT: 1: 81.5 & 2: 87.8, cogPedsQL (range scores 0-100) parent-proxy: 65.0 & child: 66.8; older age associated with better scores, longer follow-up time, ICU stay & disease severity associated with worse scores |
| Kaur et al. 2015 [29] | Children with  sepsis-associated encephalopathy | Mean 7.8 (SD 2.8) years | 35 patients, 35 healthy control children | Prospective cohort study (NOS 6/9) | 3-9 months | Malin’s Intelligence Scale for Indian Children (Indian adaptation of the WISC, intelligence) | FIQ lower in patients (100) than controls (105); VIQ lower in patients (102) than controls (109); PIQ not different in patients (98) compared to controls (101); more patients (26%) had an VIQ below average than controls (6%); FIQ 20% patients below average (controls 6%), PIQ 17% below average (controls 9%) |
| O’Brien et al. 2018 [26] | Children with moderate to severe traumatic brain injury | Mean 7.37 (SD 5.66) years | 49 | Prospective cohort study (NOS 5/9) | 12 months | Bayley-III (intellectual functioning), >30 months WPPSI (intelligence), children aged 6 and older: WISC (intelligence), vigilance subtest of the Gordon Diagnostic System (inhibitory control: executive functioning), Consonant Trigrams (working memory), Tower of London (planning: executive functioning) | Bayley-III: (scaled scores with average 10:) cognition 8, language 12, motor 10, WPPSI/WISC: FIQ 90, PIQ 88, VIQ 94, processing speed 86, inhibitory control 88, working memory 79; planning: 90 |
| Slomine et al. 2016 [23] | Children with out of hospital cardiac arrest who were comatose after ROSC (RCT: therapeutic hypothermia 33.0ºC vs. normothermia 36.8ºC) | 0-<3 years: mean 0.8 (IQR 0.6) years, 3-<6: mean 3.3 (SD 1.0) years, ≥6 years: mean 12.8 years (SD 3.8) | 85 | RCT (CCT 4/7) | 12 months | Mullen Scales of Early Learning (<6 years, intellectual functioning), Wechsler Abbreviated Scale of Intelligence (WASI, ≥6 years, intelligence) | IQ scores <6 years more than 1SD below the norm: early learning composite 81%, visual perception 70%, fine motor 71%, receptive language 71%, expressive language 79%; IQ scores ≥6 years lower than 1SD: FIQ 48%, vocabulary 53%, matrix reasoning 28% |
| Slomine et al. 2017 [20] | Children with out of hospital cardiac arrest secondary to drowning and other respiratory etiologies (RCT: therapeutic hypothermia 33.0ºC vs. normothermia 36.8ºC) | Drowning group mean 4.6 (SD 4.16) years, other: mean 5.1 (SD 5.41) years | 59 | RCT (CCT 4/7) | 12 months | Mullen Scales of Early Learning (<6 years, intellectual functioning), WASI (≥6 years, intelligence) | IQ scores drowning group; <6 years more than 1SD below the norm: early learning composite 60%, visual perception 53%, fine motor 53%, receptive language 53%, expressive language 67%; ≥6 years lower than 1SD: FIQ 90%, vocabulary 90%, matrix reasoning 70% |
| Slomine et al. 2018 [22] | Children with in-hospital cardiac arrest & broadly normal baseline neurobehavioral functioning (RCT: therapeutic hypothermia 33.0ºC vs. normothermia 36.8ºC) | At randomization: median 1 [IQR 0-4] years | 99 | RCT (CCT 4/7) | 12 months | Mullen Scales of Early Learning (<6 years, intellectual functioning), WASI (≥6 years, intelligence) | IQ scores: <6 years more than 1SD below the norm: early learning composite 74%, visual perception 63%, fine motor 63%, receptive language 68%, expressive language 65%; ≥6 years lower than 1SD: FIQ 33%, vocabulary 34%, matrix reasoning 20% |
| Slomine et al. 2018 [21] | Children with in-hospital or out of hospital cardiac arrest who needed mechanical ventilation after ROSC (RCT: therapeutic hypothermia 33.0ºC vs. normothermia 36.8ºC) | Age at follow-up: median 2.5 [IQR 1.3-6.1] years | 160 | RCT (CCT 4/7) | 12 months | Mullen Scales of Early Learning (<6 years, intellectual functioning), WASI (≥6 years, intelligence), WISC-IV or WAIS-III digit span (attention), CVLT-C (verbal memory), RCFT (visuomotor integration, memory), Controlled Oral Word Association (executive functioning), Grooved Pegboard Test (motor functioning), Beery VMI (visuomotor integration) | IQ scores medians: <6 years: early learning composite 67, visual perception 71, fine motor 72, receptive language 70, expressive language 67; ≥6 years medians: FIQ 90, vocabulary 87, matrix reasoning 93; processing speed 85; attention 90; verbal memory median range 89-96 & 100 for recognition; visual memory immediate 70, delayed 69, recognition 94; executive functioning 82; visuomotor integration 83; motor functioning 86 nondominant & 91 dominant hand |
| Wilkinson et al. 2016 [25] | Children with mild, moderate, or severe traumatic brain injury | Mean 12.5 (SD 3.5) years | 52 | Prospective cohort study (NOS 4/9) | 3, 6 and 12 months | Parent-reported Conners Third Edition Parent Rating Scale (Conners-3, attention) | Inattention elevated at 3, and 12 months following injury compared to baseline; younger age predicted attention problems 6 months following injury; 39% of the children clinically significant inattention problems at 12 months following injury, with predictors baseline levels of inattention and serum levels of the biomarker neuron-specific enolase; scale scores: inattention 60; hyperactivity/impulsivity 59; learning problems 57; executive function 58; defiance/aggression 55; peer relations 55 |

^Scores are reported as standard scores with an average of 100 and standard deviation of 15, or scaled scores with an average of 10 with standard deviation 3, or T-scores with average of 50 and standard deviation of 10. For all tests higher scores represent better performance on these tests except for the BRIEF on which higher scores represent more problems.
Abbreviations: PICU, pediatric intensive care unit; IQR, interquartile range; ECMO, extracorporeal membrane oxygenation; RCT, randomized controlled trial; NOS, Newcastle Ottawa Scale; CCT, Cochrane Collaboration’s Tool; ARHQ, Agency for Healthcare Research and Quality; IQ, intelligence quotient; FIQ, full intelligence quotient; VIQ, verbal intelligence quotient; PIQ, performance intelligence quotient; VMI, visuomotor integration; SD, standard deviation; SES, socioeconomic status^

**Supplemental table 1b. Included studies on long-term (>12 months) neurocognitive outcomes after PICU**

| *Authors* | *Study population* | *Age at PICU admission* | *Sample size* | *Study design (quality score)* | *Follow-up* | *Measurement* | *Main results* |
| --- | --- | --- | --- | --- | --- | --- | --- |
| Studies including children ≤12 months at time of PICU admission | | | | | | | |
| Eismann et al. 2020 [45] | Infants under 12 months old with abusive head trauma | 4 months (SD 2.1) | 47 | Retrospective cohort study (NOS 7/9) | 19 (SD 7) months | Mullen Scales of Early Learning | At this first assessment, no differences in scores based on patient gender, race, ethnicity, age at the time of injury, or insurance type; greater time between injury and first assessment was associated with significantly lower initial early learning composite and receptive language scores, but not with the slope of change with age; as patients aged, their early learning composite, fine motor, and expressive language scores significantly decreased; average function declined from within one SD below norms at the first assessment to over one SD below age-based norms by 3 years of age |
| Leeuwen et al. 2018 [36] | Neonatal patients treated with ECMO and/or patients with congenital diaphragmatic hernia | Congenital diaphragmatic hernia median 1 [IQR 1-2] days, ECMO other median 2 [IQR 1-4] days | 65 | Prospective cohort study (NOS 8/9) | 8 years | WISC subtests Block Design & Vocabulary (intelligence), Trail Making Test (TMT) A (processing speed), TMT B & Stroop color-word test (selective attention), Dot cancellation test (sustained attention), WISC subtest Digit Span & Immediate and delayed recall of the Rey Auditory Verbal Learning Test (RAVLT) (verbal memory), Wechsler Nonverbal Scale of Ability (WNS) subtest Spatial Span, immediate and delayed recall of the Rey Complex Figure Test (RCFT) (visuospatial memory), Behavioral Assessment of the Dysexecutive Syndrome (BADS) subtests Key Search & Modified Six Elements (executive functioning), copy of the RCFT (visuospatial integration) | IQ score within the normal range (95); sustained attention, verbal memory (immediate and delayed recall), and visuospatial memory (immediate & delayed recall, recognition) below average compared with the general population; majority of patients normal outcomes in working memory, executive functioning, and visuospatial processing (50% impaired outcomes on one or more memory and attention tests); maximum vasoactive-inotropic score (severity of illness) negatively associated with verbal and visuospatial memory at 8 years |
| Madderom et al. 2016 [13] | Neonatal patients treated ECMO | Median 28 [range 8-226] hours | 34 | Prospective cohort study (NOS 7/9) | 17-18 years | WISC (intelligence), TMT & Stroop Color-Word test (attention), subtests Rebus Learning and Auditory Comprehension of the Kaufman Intelligence Test & digit span of WAIS & Rey Auditory Verbal Learning Test (RAVLT) & Rey Complex Figure Test (RCFT) (memory), Tower Test (executive functioning), RCFT copy (visual-spatial functions), parent-reported BRIEF (executive functioning) | FIQ score 90; lower scores than normative data on short-term (*z* score = -1.40) and long-term (*z*-score = -1.54) verbal memory, short-term (*z*-score = -1.65) and long-term (z-score = -1.70) visual-spatial memory; more working memory problems (32% patients vs. 9% norm population); parent-reported executive functioning lower than normal population; no significant differences on other neuropsychological tests; multivariable regression model showed no variables as significant predictors |
| Naguib et al. 2015 [37] | Children who underwent congenital cardiac surgery within their first year of life | 3 groups of anesthetic techniques: low-dose fentanyl (LDF): mean 5 (SD 2) months, LDF + dexmedetomidine (DEX): mean 4 (SD 1) months, high-dose fentanyl: mean 4 (SD 1) months | 19 | Prospective cohort study (NOS 6/9) | ±2,5 years | Stanford-Binet Intelligence Scales - 5^th^ edition (intelligence) | HDF patients higher than the LDF+DEX group in quantitative reasoning (106 vs. 82); no other significant differences between the 3 groups; all patients compared to the general population: lower working memory (91 vs. 100), verbal IQ (88 vs. 100), FIQ (91 vs. 100); no differences with norm on PIQ, visual spatial processing, knowledge score, quantitative reasoning; longer ICU stay negatively associated with FIQ; stress response markers positively associated with VIQ |
| Ryerson et al. 2015 [32] | Survivors of cardiac Extracorporeal Life Support (ECLS) | Median 2.1 [IQR 0.4-7.1] months | 50 | Prospective cohort study (NOS 7/9) | Mean 43.4 (SD 14.5) months | WPPSI (intelligence), Beery VMI (visuomotor integration) | Survivors without chromosomal abnormalities (44 children): FIQ 80, PIQ 81, VIQ 82, VMI 81; 25% >2 SD below population mean; survivors with chromosomal abnormalities (6 children): 100% >2 SD below population; multiple linear regression: higher weight associated with higher FIQ, PIQ, and VIQ scores; for VIQ: higher SES associated with higher scores |
| Sadhwani et al. 2019 [40] | Patients treated with ECMO for cardiac indications before the age of 36 months | Median 5.5 [IQR 3-20] days | 28 | Retrospective cohort study (NOS 7/9) | Median 19 [IQR 13-27.5] months | Bayley-III (intellectual functioning) | Cognition 93, Language 88, motor 80; older age, more cardiac catheterization procedures & more cardiac operations at ECMO associated with lower scores |
| Schiller et al. 2016 [38] | Neonatal patients treated with ECMO | Median 1 [range 0-23] day | 178 | Prospective cohort study (NOS 7/9) | 2, 5 and 8 years of age | Bayley Developmental Scales (BOS, 2-year-olds, intellectual functioning), Revised Amsterdam Child Intelligence Test short-form (5-year-olds, intelligence), WISC (8-year-olds) (intelligence), Dot Cancellation paper & pencil test (selective attention) | Intelligence within normal range and stable at 2, 5, and 8 years of age; at 8 years of age: 3% low IQ scores (<70), 22% below-average (≤85), 58% average (85-115), 17% above-average (≥115); 37% needed extra help at school, 7% attended special education; selective attention better in regular education than children needing extra help or special education; patients with congenital diaphragmatic hernia lower IQ than other diagnoses, IQ scores range between 93 and 104 between diagnoses and ages, selective attention at 8 years of age range z= -1.39 and z= -3.48 |
| Schiller et al. 2017 [46] | Neonatal patients treated with ECMO | Mean 20 (SD 24) hours | 23 patients, 54 healthy control children | Prospective cohort study (NOS 7/9) | Mean 11.9 (SD 2.6) years | Subtests of the Neuropsychological Assessment (NEPSY-II) test battery: children aged 8-12 years 9 subtests, older children 6 subtests (attention, executive functioning, memory, visuospatial integration & language) | Altered white matter integrity and smaller bilateral hippocampal volume associated with lower verbal memory performance (only associations reported, no test results) |
| Schiller et al. 2017 [42] | Neonatal patients treated with ECMO and/or with congenital diaphragmatic hernia with IQ ≥80 & memory impairment | Median 2 [IQR 1-3] days | 38 | Prospective cohort study (NOS 6/7) | 8-12 years | WISC subtests Block Design & Vocabulary (intelligence), Dot cancellation test (sustained attention), TMT B & Stroop color-word test (selective attention), TMT A (processing speed), WISC subtest Digit Span & Wechsler Nonverbal Scale of Ability (WNS) subtest Spatial Span (working memory), RAVLT (verbal memory), RCFT (visuomotor integration), Behavioral Assessment of the Dysexecutive Syndrome (BADS) subtests Key Search & Modified Six Elements (executive functioning) | Patients average IQ (FIQ = 100) compared with the general population, lower on immediate (z= -1.58), delayed (z= -1.90) and recognition (z= -1.19) verbal memory & immediate (z= -1.65), delayed (z= -1.65), and recognition (z= -0.63) visuospatial memory tasks, lower sustained attention (z= -1.13); not significantly lower than the norm on selective attention (z= -.31), processing speed (z= -0.33), executive functioning (z= 0.01 & -0.08), and visuomotor integration (z= 0.14) |
| Schiller et al. 2018 [14] | Neonatal patients treated with ECMO and/or with congenital diaphragmatic hernia with IQ ≥80 & memory impairment (RCT: Cogmed Working Memory Training 45 minutes, 5 days a week, 5 weeks vs. no intervention) | Median 2 [IQR 1-3] days | 43 (20 patients intervention group, 25 patients control group) | RCT (CCT 2/7) | Immediately & 1 year after Cogmed Working Memory Training (age at training mean 10 (SD 2) years | Parent-reported BRIEF (executive functioning), WISC-III subtest Digit Span & WNV subtest spatial span (working memory), Dot cancellation test (sustained attention), TMT B & Stroop color-word test (selective attention), TMT A (processing speed), RAVLT (verbal memory), RCFT (visuospatial memory), Behavioral Assessment of the Dysexecutive Syndrome (BADS) subtests Key Search & Modified Six Elements (executive functioning) | Parent-reported total score executive functioning higher 1 year after Cogmed training; verbal working memory & visuospatial working memory improvements directly after Cogmed Working Memory Training compared to the control group, but not after 1 year; after 1 year only long-term visuospatial memory improvements compared to the control group; attention, processing speed, executive functioning, and visuospatial processing were similar directly after and 1 year after Cogmed training in both groups |
| Schiller et al. 2019 [44] | Neonatal patients treated with ECMO and/or with congenital diaphragmatic hernia with IQ ≥80 & memory impairment (RCT: Cogmed Working Memory Training 45 minutes, 5 days a week, 5 weeks vs. no intervention) | Median 1 [IQR 1-2] day | 34 (15 patients intervention, 19 patients control group) | RCT (CCT 2/7) | Immediately & 1 year after Cogmed Working Memory Training (age at training mean 10 (SD 2) years | Digit Span of the WISC-III (verbal working memory), Spatial Span of the Wechsler Nonverbal Scale of Ability (visuospatial working-memory), RCFT (visuospatial memory) | Children in the CWMT who improved with more than 1SD on verbal working-memory from baseline to post-treatment had significantly higher fractional anisotropy in the left superior longitudinal fasciculus post-treatment; range z-scores on memory tasks control group: -2.08 – 0.43, intervention group -1.71 – 1.27 |
| Sterken et al. 2016 [39] | Children with congenital heart disease who underwent heart surgery | Median 0.16 [IQR 0.04-0.4] years | 107 patients, 77 healthy control children | Prospective cohort study (NOS 8/9) | 4 & 7 years | WPPSI, Beery VMI, Baseline Speed (attention), Memory Search Objects, Response Organization Arrows (executive functioning), Tapping (motor functioning) | IQ scores lower in patients (FIQ 96, VIQ 98, PIQ 95) than controls (FIQ 103, VIQ 103, PIQ 102); difference of 12 IQ points between both groups at 4-years follow-up decreased to 7 IQ points at 7-years follow-up; visuomotor integration 9; attention, motor functioning and executive functioning, lower in patients than controls, working memory no differences; inhibition reaction time improved in both study groups at 7-years follow-up & did not differ between both groups from 4-years follow-up to 7-years follow-up; deficits between 4 & years follow-up for VMI, alertness, motor coordination not worse for patients with CHD at 7-years follow-up than healthy controls |
| Trieu et al. 2017 [41] | Neonates with tetanus | Age at follow-up: median 36 [IQR 30-59] months | 17 patients, 18 healthy control children | Prospective cohort study (NOS 6/9) | 2-5 years | Bayley-III (<42 months, intellectual functioning), Movement Assessment Battery for Children (motor functioning) | Intellectual functioning 6; Bayley fine motor score 6 & gross motor 8; Movement Assessment Battery: 70; no significant differences total scores patients vs. healthy control children but trend toward lower scores in patients; more patients with fine motor scores <2 SD than controls, patients with severe disease lower scores than mild disease |
| Urschel et al. 2018 [31] | Children who underwent heart transplantation ≤4 years of age | Cardiomyopathy group: median 0.82 [IQR 0.48-2.35] years, congenital heart disease group: median 0.7 [IQR 0.28-2.95 years] | 55 | Prospective cohort study (NOS 7/9) | >12 months | WPPSI (intelligence), Beery VMI (visuomotor integration) | FIQ, VIQ, PIQ, and VMI below population norms and lower in children with congenital heart disease (CHD) than children with cardiomyopathy (CMP); CHD FIQ 75, PIQ 74, VIQ 78, VMI 75; CMP FIQ 86, PIQ 86, VIQ 89, VMI 82; lower FIQ and VMI associated with higher creatinine and lactate pre-transplantation, longer stay in intensive care and lower socioeconomic status |
| Van den Bosch et al. 2015 [43] | Neonatal patients treated with ECMO | Median 0 [range 0-16] days | 36 patients, 64 healthy control children | Prospective cohort study (NOS 7/9) | Mean 11.1 years | Subtests of the NEPSY-II test battery: children aged 8-12 years 9 subtests, older children 6 subtests (attention, executive functioning, language, memory, sensorimotor functioning, visuospatial integration) | Patients worse scores on verbal memory test & visuomotor integration test compared with healthy control children; on other subtests lower scores but no significant differences between groups |
| Varghese et al. 2018 [33] | Neonates with encephalopathy | Neonates during the first 72 hours of life | 19 patients, 19 healthy control children | Retrospective cohort study (NOS 6/9) | 6-8 years | Bender Gestalt II test (visual motor integration), Malin’s Intelligence Scale for Indian Children (Indian adaptation of the WISC, intelligence) | Visual motor and perception functions significantly lower in patients (67) compared to controls (94); significant lower IQ scores in patients versus controls: FIQ patients mean 75 vs. controls 117, VIQ patients 84 vs. controls 122, PIQ patients 65 vs. controls 113 |
| Vassel-Hitier et al. 2019 [34] | Children with moderate to severe traumatic brain injury before the age of 18 months | Mean 0.7 (SD 0.5) years | 21 | Prospective cohort study (NOS 6/9) | Mean 6.8 (SD 1.8) years | WPPSI-III & WISC-IV (intelligence) | FIQ 77, VIQ 80, PIQ 84, processing speed 83; VIQ <80 in 57% & PIQ 76%; negative long-term consequences of young age at injury on cognitive and language development |
| Wagenaar et al. 2019 [35] | Infants with perinatal arterial ischemic stroke | Gestational age mean 40.4 weeks (SD 1.4) | 44 | Retrospective cohort study (NOS 7/9) | Median 24.0 (IQR 18.5-24.4) months | Griffiths Mental Development Scale & Bayley-III (intellectual functioning) | 9 infants developed cerebral palsy and 8 cognitive deficit (<1SD); large (versus small) lesions were associated with increased risk of an adverse outcome, including cerebral palsy and cognitive deficit |
| Studies including children >12 months at time of PICU admission | | | | | | | |
| Abend et al. 2015 [50] | Children with acute neurologic conditions | Median 10.6 [IQR 6.7-15.4] years | 20 | Prospective cohort study (NOS 4/9) | Median 2.6 [1.2–3.8] years | Parent-reported Behavior Rating Inventory of Executive Function (BRIEF, executive functioning) | Electrographic status epilepticus (ESE) and electrographic seizures (ES) no worse scores compared to children without seizures; BRIEF composite score clinically significant high (73, mean 50) in children with ESE |
| Afshar et al. 2018 [48] | Children who underwent liver transplantation | Mean 2.50 (SD 3.15) years | 40 | Cross-sectional study  (ARHQ 4/11) | Mean 8.71 (SD 3.82) years after transplantation | Wechsler Intelligence Scale for Children Fourth Edition (WISC- IV, intelligence) & Wechsler Individual Achievement Test Second Edition (WIAT- II, intellectual functioning) | Compared with normative data scores significantly lower on: full-scale IQ (FIQ) 93, Verbal Comprehension Index 93, Perceptual Reasoning Index 94, Working Memory Index 92, Numerical Operations 91, Mathematical Reasoning 93, Mathematics Composite 92; scores comparable to the norm on: Processing speed 100, Word Reading 100, Phonological Decoding 100, Spelling 101 |
| Eder et al. 2020 [49] | Children who underwent liver transplantation | Age at follow-up median 9.6 (range 6.0-16.9) years | 36 | Prospective cohort study (NOS 6/9) | Median 9.6 (range 1.8-8.3) years | WISC-IV | Patients without cognitive impairment (IQ ≥ 70) scored within the lower normal range (mean 87, SD 16) in all indices and the total IQ; compared to the normal population mean, patients scored significantly lower in all indices |
| Jacobs et al. 2020 [54] | Critically ill children admitted to the PICU with an expected stay of >24 hours (RCT: No parenteral nutrition for up to 1 week in the PICU, when enteral nutrition was insufficient vs. full nutrition up to caloric targets with supplemental parenteral nutrition) | Age at follow-up mean 7.3 (SD 4.3) years | 684 patients, 369 healthy control children | RCT (CCT 4/7) | 4 years | Parent-reported BRIEF (executive functioning), Wechsler intelligence scales (WPPSI, 2,5-5 years, WISC 6-16 years, WAIS ≥17 years, intelligence), Beery VMI (visuomotor integration), Amsterdam Neuropsychological Tasks: Baseline Speed (attention), Tapping (motor functioning), CMS (memory) | Patients worse scores than controls on parent-reported executive functioning (range scales 48.9-50.6), FIQ 93, VIQ 95, PIQ 93, visuomotor integration 9, alertness, verbal & nonverbal memory, working memory; intervention groups: late parenteral nutrition at the PICU no differences on neurocognitive outcomes |
| Meyburg et al. 2018 [51] | Children with postoperative delirium  following major surgery (operation electively planned) | Age at follow-up mean 5.1 (SD 4.6) years | 47 | Prospective cohort study (NOS 7/9) | Mean 17.7 (SD 2.9) months | Bayley-III (intellectual functioning, WPPSI-III & WISC-IV (intelligence) & parent-reported BRIEF (executive functioning) | Cognitive performance in the normal range, mean cognitive score lower than the normal population (11.2 points lower) & proportion of patients with cognitive delay higher than in the normal population; cognitive performance negatively associated with length of PICU stay; lower global cognitive functions associated with worse executive functions; multivariate analyses: higher premorbid developmental/medical risk significant predictor for lower cognitive outcomes |
| Verstraete et al. 2016 [52] | Critically ill children admitted to the PICU with an expected stay of >24 hours | Mean 3.8 (SD 3.3) years | 449 patients, 100 healthy control children | Prospective cohort study (NOS 8/9) | 4 years | Wechsler intelligence scales (WPPSI, 4-5 years, WISC 6-16 years, WAIS ≥17 years, intelligence), Beery VMI (visuomotor integration), Amsterdam Neuropsychological Tasks: Baseline Speed (attention), Response Organization Objects (executive functioning), Tapping (motor functioning), CMS (memory) | Development cohort (stable potentially harmful threshold levels of exposure to circulating di(2-ethylhexyl)phthalate metabolites identified): FIQ 88, visuomotor integration 9, memory 8, working memory 9; validation cohort (associations between phthalate exposure during PICU stay and the neurocognitive scores validated): FIQ 85, visuomotor integration 8, memory 8, working memory 9; exceeded thresholds of circulating di(2-ethylhexyl)phthalate associated with attention deficit and impaired motor coordination in patients vs. healthy controls |
| Verstraete et al. 2019 [53] | Critically ill children admitted to the PICU with an expected stay of >24 hours (RCT: No parenteral nutrition for up to 1 week in the PICU, when enteral nutrition was insufficient vs. full nutrition up to caloric targets with supplemental parenteral nutrition) | Age at follow-up: mean 5.7 (SD 4.5) years | 786 patients, 405 healthy control children | RCT (CCT 4/7) | 2 years | Parent-reported BRIEF (executive functioning), Wechsler intelligence scales (WPPSI, 2,5-5 years, WISC 6-16 years, WAIS ≥17 years, intelligence), Beery VMI (visuomotor integration), Amsterdam Neuropsychological Tasks: Baseline Speed (attention), Response Organization Objects (executive functioning), Tapping (motor functioning), CMS (memory) | Patients worse scores than controls on parent-reported executive functioning (range scales 49.9-51.4), FIQ 91, VIQ 92, PIQ 92, visuomotor integration 8, alertness, verbal & nonverbal memory, working memory; intervention groups: late parenteral nutrition at the PICU better than early parenteral nutrition on visuomotor integration, and subscales of the parent-reported executive functioning; use of benzodiazepines and corticosteroids associated with poorer outcomes; α2 agonists associated with better overall executive functioning and visuomotor integration |
| Volpe et al. 2017 [47] | Children with mild, moderate, or severe traumatic brain injury | Median 6 [range 1-2] years | 25 | Prospective cohort study (NOS 5/9) | Median 6 years (range 2-9 years) | WISC-IV (intelligence) | Classification based on neurological outcome: good recovery 60%, disability 40%; all subscales and FIQ lower in disability group compared to good recovery group; 40% of children with traumatic brain injury neuropsychological impairment; working memory good recovery 94, disability 74; perceptual reasoning good recovery 96, disability 75; verbal comprehension good recovery 84, disability 65; processing speed good recovery 97, disability 74; FIQ good recovery 87, disability 65 |

^Scores are reported as standard scores with an average of 100 and standard deviation of 15, or scaled scores with an average of 10 with standard deviation 3, or T-scores with average of 50 and standard deviation of 10. For all tests higher scores represent better performance on these tests except for the BRIEF on which higher scores represent more problems.
Abbreviations: PICU, pediatric intensive care unit; IQR, interquartile range; ECMO, extracorporeal membrane oxygenation; RCT, randomized controlled trial; NOS, Newcastle Ottawa Scale; CCT, Cochrane Collaboration’s Tool; ARHQ, Agency for Healthcare Research and Quality; IQ, intelligence quotient; FIQ, full intelligence quotient; VIQ, verbal intelligence quotient; PIQ, performance intelligence quotient; VMI, visuomotor integration; SD, standard deviation; SES, socioeconomic status^

**Supplemental table 2a. Included studies on short-term (≤12 months) health-related quality of life outcomes after PICU**

| *Authors* | *Study population* | *Age at PICU admission* | *Sample size* | *Study design (quality score)* | *Follow-up* | *Measurements reported by the parent/child* | *Main results* |
| --- | --- | --- | --- | --- | --- | --- | --- |
| Studies including children ≤12 months at time of PICU admission | | | | | | | |
| Bembea et al. 2020 [15] | Children who underwent ECMO for more than 24 hours for any indication | Median 35 [2 days – 20 months] days | 40 | Prospective cohort study (NOS 8/9) | 6 & 12 months | Parent: ITQOL (<2 years), PedsQL (≥2 years) | 32 patients <2 years median 85 [IQR 60-85] (1 SD below general population), 8 patients ≥2 years median 69 [IQR 46-83]; scores are below general population means |
| Meert et al. 2018 [55] | Children with in-hospital cardiac arrest | Children <5 years: 0.4 [IQR 0.1-1.3] years, children >5 years: 11.5 [IQR 9.0-14.3] years | 138 | Prospective cohort study (NOS 3/9) | <24 hours of admission, 3 & 12 months | Infant Toddler Quality of Life Questionnaire (ITQOL) <5 years subscales parent impact-emotion & parent impact-time, Child Health Questionnaire (CHQ) >5 years subscales parent impact-emotion, parent impact-time & family activities | Caregiver burden improved at 12 months compared to pre-arrest & 3 months; both age groups: larger caregiver burden at all times compared to reference values; worse neurobehavioral functioning at 3 months associated with larger burden at 12 months |
| Vet et al. 2016 [56] | Children who required mechanical ventilation (RCT: daily sedation interruption in addition to protocolized sedation vs. protocolized sedation only) | Median 2.3 [IQR 1.0-7.9] months | 64 parents, 4 children | RCT (CCT 4/7) | 8 weeks | Parent: CHQ Infant Toddler – 97 (CHQ-IT97) 0-3 years, CHQ Parent Form – 50 (CHQ-PF50) 4-18 years  Child 12-18 years: CHQ Child Form – 87 (CHQ-CF87) | Daily sedation interruption + procotolized sedation vs. protocolized sedation: no differences; patients lower scores than normative data; general behavior higher; self-reports no differences with normative data |
| Studies including children >12 months at time of PICU admission | | | | | | | |
| Aspesberro et al. 2016 [57] | Critically ill children admitted to the Pediatric and Cardiac ICU | Mean 5.6 (SD 6.1) years | 263 | Prospective cohort study (NOS 6/9) | <24 hours after discharge & 4–12 weeks | Parent: PedsQL 4.0  Children: PedsQL >8 years | Improvement scores highest on physical domain compared to psychosocial domain; patients with nonchronic disease 86.9, complex chronic 80.6, and noncomplex chronic 85.4 disease at follow-up |
| Biagas et al. 2020 [67] | Critically ill children admitted to the PICU with hyperglycemia (RCT: lower-target vs. higher target glycemic control) | Median 10.1 [IQR 5.1-14.1] years | 214 | RCT (CCT 4/7) | First week of admission & 1 year | Parent: PedsQL | Higher target group: mean total score improved from 67.7 (20.9) at baseline to 76.4 (20.5) at 1 year (ascribed to improvement in psychosocial health score), improvement in higher target group was greater than in the lower target group |
| Boeschoten et al. 2020 [68] | Children with severe acute asthma | Median 8 [IQR 6-12] years at follow-up | 50 | Prospective cohort study (NOS 7/9) | 5 months (range 1-12 months) | Child: Asthma-related quality of life (PAQLQ), Visual Analog Scale (VAS) | No differences QoL between children admitted to the PICU and general ward |
| Choong et al. 2018 [58] | Critically ill children admitted to PICU for at least 48 hours with one or more organ dysfunction | Median 7.2 [IQR 2.9-13.4] years | 6 | Prospective cohort study (NOS 4/9) | 3 & 6 months | Parent & child: KIDSCREEN (child >8 years) | Physical well-being, psychological well-being and social support domains most affected and below the referenced population 3 and 6 months post PICU discharge |
| Colville et al. 2019 [59] | Critically ill children admitted to the PICU and without significant learning disabilities | Median 11 (range 7-17) years | 97 | Prospective cohort study (NOS 3/9) | 3 months | Child: PedsQL Multidimensional Fatigue Scale 7-17 years | Total fatigue scores no significant differences PICU survivors (79.6) and normative data (81.8); 9% scored more than 2 SD below norms; cognitive fatigue subscale significantly lower in PICU survivors than normative data |
| Ebrahim et al. 2015 [65] | Children urgently admitted to the PICU | Mean 10.3 (SD 4.6) years | 52 | Prospective cohort study (NOS 3/9) | <24 hours of admission & 1 month | Parent: HUI-3 and Visual Analog Scale (VAS) | VAS scores (direct measurement) greater than HUI-3 utility scores (indirect measurement, score between 0 and 1) at baseline (VAS 72.5; HUI3 0.70); & 1 month (VAS 70.0; HUI3 0.58); no differences between adolescents & nonadolescents or reasons for admission |
| Fink et al. 2019 [64] | Children with traumatic or nontraumatic brain insult (RCT: early protocolized rehabilitation within 72 hr PICU vs. usual care) | Mean 10.8 (95% confidence interval 9.7-11.9) years | 58 | RCT (CCT 3/7) | Baseline & 6 months | Parent: PedsQL | Quality of life was worse 6 months later compared to premorbid baseline; no differences between early protocolized rehabilitation (premorbid: 91, 6 months: 78) and usual care (premorbid: 86, 6 months: 74) |
| Hordijk et al. 2019 [60] | Critically ill children admitted to the PICU with an expected stay of >24 hours | Median 1.3 (IQR 0.2-5.6) years | 576 | Prospective cohort study (NOS 6/9) | 6 months | Parent: ITQOL 0-3 years, CHQ-PF50 4-18 years | Parent-reported HRQoL lower than healthy children; older age, longer length of stay & more severe illness associated with less change in health & physical functioning; cardiac surgery associated with better scores on physical functioning & change in health; parents̕ mental HRQoL correlated with HRQoL they reported for their child |
| Meert et al. 2020 [69] | Children with community-acquired septic shock without preexisting severe mental disability | 0–12 months: 22%  13-24 months:  12%  2-4 years:  16%  5-7 years:  11%  8-12 years:  17%  13-17 years:  23% | 204 | Prospective cohort study (NOS 5/9) | 1 week; 1, 3, 6 and 12 months | Parent: PedsQL | Physical and psychosocial HRQoL lower than reference norms during the first year, many recovered to baseline; risk factors for poor HRQoL were neurologic complications during the hospitalization and dependence on a medical device 1 month postadmission |
| Meert et al. 2020 [70] | Children with community-acquired septic shock with preexisting severe mental disability | 0–12 months: 10%  13-24 months:  9%  2-4 years:  20%  5-7 years:  10%  8-12 years:  22%  13-17 years:  29% | 121 | Prospective cohort study (NOS 5/9) | 1 week; 1, 3, 6 and 12 months | Parent: Stein-Jessop Functional Status II-R Short Form (FSII-R) | HRQoL below baseline during the first year; single parent families increased risk; total FSII-R scores significantly declined from preadmission baseline to day 7; total FSII-R scores showed some improvement by month 1 |
| Nelson et al. 2020 [71] | Children aged 8-17 years admitted to the PICU or cardiothoracic ICU with an expected stay >24 hours | Mean 12.9 (range 8-17) years | 111 | Prospective cohort study (NOS 4/9sil) | 72 hours | Parent and child: PedsQL | Significant impairments on all domains of HRQoL both child report and parent-report; no significant differences between children with previous PICU admissions or those who had not previously been admitted |
| Peña et al. 2018 [66] | Severely burned children (RCT: day hospital-based exercise program (DAYEX) vs. community-based exercise program (COMBEX)) | Mean 14.4 (SD 3.8) years in DAYEX & mean 13.2 (SD 2.9) years in COMBEX) | 18 | RCT (CCT 0/7) | Discharge & 1 year | Parent: CHQ-PF28  Child: CHQ-CF87 | Both groups improvements for physical functioning at 1 year postburn both parent- and child-reported; parents reported more improvement in role/social limitations-emotional, bodily pain, and family activities in the COMBEX group than the DAYEX group; children’s perspective more improvement in family cohesion in the COMBEX group than the DAYEX group (no comparison with normative data/healthy children) |
| Silver et al. 2020 [72] | Children younger than 5 years admitted to the PICU and who experienced delirium | Median 13 [IQR 5-29] months | 207 | Prospective cohort study (NOS 4/9) | 1 and 3 months | Parent: ITQOL | HRQoL of PICU patients overall consistently lower than age-related norms; children with delirium scored lower in every quality of life domain compared with children without delirium |
| Ward et al. 2017 [61] | Children with acute respiratory distress syndrome | Median 64 [IQR 38-145] months | 23 parents, 7 children | Prospective cohort study (NOS 4/9) | Mean 10.7 (SD 3.9) months | Parent: CHQ parent form  Child: when developmentally appropriate: CHQ child form | Parent reports significantly lower physical functioning, role limitations physical, and general health perceptions compared to normative data; children reported better scores compared with parents; parents reported diminished mental health for children of increasing grade level; parents with higher education reported lower bodily pain |
| Watson et al. 2018 [62] | Children with acute respiratory failure (RCT: team-based, nurse-implemented, and goal-directed sedation protocol vs. usual care) | Median 1.4 [IQR 0.3-6.8] years in the sedation protocol group, median 3.4 [IQR 0.8-8.9] years in the usual care group | 838 parents, 105 children | RCT (CCT 2/7) | Median 6.9 [5.7–8.5] months | Parent: ITQOL-97 <2 years, PedsQL ≥2 years  Child: PedsQL | No significant differences in total scores between treatment arms; no significant differences in scores between parent- and self-report; across all age groups, parents reported  HRQoL scores lower than the norm; scores on temperament and moods & general behavior higher than the norm |
| Watson et al. 2019 [63] | Children with acute respiratory failure (RCT: team-based, nurse-implemented, and goal-directed sedation protocol vs. usual care) | 52% <2 years, 18% 2-<6 years, 30% 6-<18 years | 949 | RCT (CCT 2/7) | 6 months | Parent: ITQOL-97 <2 years, PedsQL ≥2 years | ITQOL: Patients with normal baseline function worse than norms on physical abilities, growth & development, pain & discomfort, getting along with others, and general health perceptions, better on general behavior; patients with impaired baseline function worse than norms on all domains except general behavior; PedsQL: normal baseline function lower scores for emotional functioning & higher social functioning; patients with impaired baseline function lower than norms on all subscales; 19% had impaired HRQoL; impaired HRQoL associated with older age, non-white or Hispanic race, cancer & inadequate sedation management |
| Zimmerman et al. 2020 [73] | Critically ill children with community-acquired septic shock requiring vasoactive-inotropic support | Median 6.4 [IQR 1.7-13.0] years | 364 | Prospective cohort study (NOS 6/9) | 1 week; 1, 3, 6 and 12 months | Parent: PedsQL or FSII-R | Magnitude and duration of organ dysfunction, need for organ failure rescue, duration of hospitalization, and older age were risk factors associated with deterioration of HRQoL at 3 months |
| Zimmerman et al. 2020 [74] | Critically ill children with community-acquired septic shock requiring vasoactive-inotropic support | Mean 7.4 (SD 5.8) years | 389 | Prospective cohort study (NOS 6/9) | 1 week; 1, 3, 6 and 12 months | Parent: PedsQL or FSII-R | 50% of patients had deterioration of HRQoL after 1 week, 37% after 1 months, 30% after 3 months, 35% of survivors had HRQoL deterioration that persisted at least 1 year following hospitalization |

^Scores for HRQoL questionnaires range from 0-100, unless otherwise indicated. For all questionnaires higher scores represent better performance.
Abbreviations: HRQoL, health-related quality of life; PICU, pediatric intensive care unit; IQR, interquartile range; ECMO, extracorporeal membrane oxygenation; RCT, randomized controlled trial; NOS, Newcastle Ottawa Scale; CCT, Cochrane Collaboration’s Tool; ARHQ, Agency for Healthcare Research and Quality; SD, standard deviation; SES, socioeconomic status^

**Supplemental table 2b. Included studies on long-term (>12 months) health-related quality of life outcomes after PICU**

| *Authors* | *Study population* | *Age at PICU admission* | *Sample size* | *Study design (quality score)* | *Follow-up* | *Measurements reported by the parent/child* | *Main results* |
| --- | --- | --- | --- | --- | --- | --- | --- |
| Studies including children ≤12 months at time of PICU admission | | | | | | | |
| Campbell et al. 2018 [75] | Children with perinatal asphyxia encephalopathy (RCT: intensive care plus total body cooling for 72 hours vs. intensive care without cooling) | Age at follow-up: hypothermia group median 6.3 [IQR 6.1-6.8] years, control group standard care median 6.3 [IQR 6.1-6.7] years | 145 (75 hypothermia group, 70 control group) | RCT (CCT 4/7) | 6-7 years | Parent: Health Utilities Index 2 & 3 (HUI-2 & HUI-3) | Speech most affected compared with other aspects of HRQL; normal emotional functioning was similar in both groups; mean HUI3 scores 0.73 in the hypothermia group and 0.62 in the control group; no significant differences between intervention groups |
| Di Leo et al. 2019 [76] | All patients who were treated with ECMO | Age at follow-up: median 20.96 (range 13.33-35.58) years | 20 | Prospective cohort study (NOS 4/9) | Median 20.75 (range 11.50-24.08) years | Child: PedsQL 4.0 (adolescent version 13-18 years, adult version 18-25 years old) | Both adolescents & adults lower scores in patients (total: 81.3, physical: 85.2, psychosocial 79.2) than normative data (total: 83.9, physical: 87.8, psychosocial: 81.8); differences not significant; 90% no disability |
| Eismann et al. 2020 [45] | Infants under 12 months old with abusive head trauma | 4 months (SD 2.1) | 27 | Retrospective cohort study (NOS 7/9) | 19 months (Sd 7) | Parent: PedsQL | Average physical health 86.8, average psychosocial health 85.2 and total HRQoL score 85.9 and are slightly higher than the normative scores for the healthy population; 8 patients at risk for impaired HRQoL; scale scores not different between gender, race age at time of injry, age at assessment, injury severity or early therapy participation; biological parents rated children better than foster/adoptive caregivers |
| Elias et al. 2017 [77] | Patients with cardiac disease treated with ECMO | Median 63.5 [IQR 1-768] days | 33 | Prospective cohort study (NOS 4/9) | Median 7.3 years | Parent: PedsQL 4.0  Child 2-18 years old: PedsQL 4.0 | Children self-reported (<13 years, total: 63.6, psychosocial: 63.5, physical 63.9) & proxy-reported scores (total: 68.3, psychosocial: 67.2, physical: 70.3) both significantly lower than normative total; adolescent (13-18 years) self-reported (total: 79.5, psychosocial: 79.5, physical: 84.1) and proxy-reported (total: 73.3, psychosocial 71.3, physical 77.3) scores not significantly lower than normative total scores |
| Fleck et al. 2017 [78] | Children who required mechanical circulatory support | Median 2 [IQR 0-213] months | 19 | Prospective cohort study (NOS 4/9) | Median 4.5 [IQR 0.3-11.3] years | Parent: KINDL  Child >12 years: Short-Form-36 (SF-36) Health Survey self-report | Parent-reported HRQoL lower (56) than healthy children; self-reported HRQoL of patients (physical: 81.0, social: 100.0, emotional role limitation: 91.8, mental health: 81.6) comparable to healthy children (physical: 94.2, social: 89.4, emotional role limitation 93.0, mental health: 76.4) |
| Madderom et al. 2016 [13] | Neonatal patients treated with ECMO | Median 28 [range 8-226] hours | 30 | Prospective cohort study (NOS 7/9) | 17-18 years | Child: PedsQL self-report | No significant differences patients & healthy controls: physical functioning: 0.25, emotional functioning: 0.49, social functioning: 0.06, school functioning −0.01, psychosocial functioning: 0.24 |
| Öst et al. 2018 [79] | Children with congenital diaphragmatic hernia | Gestational age mean 38 (SD 2) weeks | 35 | Prospective cohort study (NOS 5/9) | Median 13 [IQR 6-20] years | Child & parent: KIDSCREEN-52 | Patients scored higher than normative data on self-perception, autonomy, parent relations and home life, financial resources, & school environment & on other domains comparable to normative data; parent reports were similar to children’s own scores |
| Polić et al. 2017 [82] | Critically ill children admitted to the PICU without metabolic or other genetic disorder, congenital malformations or any syndrome | Neonates (reported separately for pre-term and full-term born) | 131 | Prospective cohort study (NOS 6/9) | 6-12 years | Parent: Royal Alexandra Hospital for Children Measure of Function (RAHC MOF) | Total score in term-born ICU children 85.0 (scores between 71-100 reflect minimal health problems and good QoL); 6.9% a fair QOL, 93.1% good QOL; need of mechanical ventilation associated with worse QOL compared to no need for mechanical ventilation |
| Schiller et al. 2018 [14] | Patients treated with ECMO and/or with congenital diaphragmatic hernia with IQ ≥80 & memory impairment (RCT: Cogmed Working Memory Training 45 minutes, 5 days a week, 5 weeks vs. no intervention) | Median 2 [IQR 1-3] days | 43 (20 patients intervention group, 25 patients control group) | RCT (CCT 2/7) | Immediately & 1 year after Cogmed Working Memory Training | Parent: Pediatric Quality of Life Inventory (PedsQL) & CHQ  Child: PedsQL | Self-reports and parent-reports of QOL immediately and 1 year after Cogmed training were not different from baseline QOL (no comparison with normative data/healthy children) |
| Van Zellem et al. 2015 [80] | Children admitted to the PICU after cardiac arrest | Median 4.9 [range 0-193] months | 57 | Prospective cohort study (NOS 5/9) | Median 5.6 [1.8-11.0] years | Parent: HUI 2 & 3, CHQ-IT97 0-3 years, CHQ-PF50 4-18 years  Child 12-18 years: CHQ-CF87 | HUI scores lower in patients than normative data; CHQ significantly lower on role functioning physical, general health perceptions, parental impact emotional, higher on family cohesion; self-reports no differences with normative data |
| Yu et al. 2018 [81] | All patients treated with ECMO | Median 1 [IQR 0-77] day | 47 | Retrospective cohort study (NOS 3/9) | Median 4.9 [IQR 3.1-7.9] years | Parent: PedsQL | Average overall QOL score for patients 70.6; 28% of patients had overall QOL scores equal or greater to the average score; 43% had QOL score more than 1SD below normative data; neurologic complications (particularly intracranial ischemia or hemorrhage) negatively associated with QOL |
| Studies including children >12 months at time of PICU admission | | | | | | | |
| Chandler et al. 2015 [84] | Children older than 30 days who received extracorporeal life support (ECLS) | Median 2.5 [0.97-9.64] years | 16 | Prospective cohort study (NOS 4/9) | Median 4.4 [IQR 2.5-5.8] years | Parent & child: PedsQL 4.0 | Mean total PedsQL scores 73.9 (31% <1SD); physical 79.7 (25%<1SD); psychosocial 70.9 50% <1SD); 70% normal quality of life; no clinical characteristics correlated with PedsQL scores |
| Ekinci et al. 2017 [85] | Children with moderate to severe traumatic brain injury and ADHD (postinjury) with normal intelligence | Mean 12.7 (SD 3.1) years | 20 patients with traumatic brain injury & ADHD, 20 control children with ADHD | Cross-sectional study (ARHQ 6/11) | 1-4 years | Parent: Kinder Lebensqualitätsfragebogen: Children’s Quality of Life Questionnaire-Revised (KINDL-R) | KINDL-R total score (patients 83.4, control 88.9), emotional well-being & self-esteem significantly lower in patients versus control; physical comparable scores in patients vs. controls |
| Hordijk et al. 2020 [83] | Critically ill children admitted to the PICU with an expected stay of >24 hours (RCT: No parenteral nutrition for up to 1 week in the PICU, when enteral nutrition was insufficient vs. full nutrition up to caloric targets with supplemental parenteral nutrition) | Mean 5.7 (SD 0.2) years | 786 patients, 405 healthy control children | RCT (CCT 3/7) | 2 years | Parent: ITQOL 0-3 years, CHQ-PF50 4-18 years | Lower scores for patients vs. healthy control children; in younger children patients lower growth and development scores, in older children lower role functioning and mental health than healthy control children; no differences in scores between intervention groups |
| Jain et al. 2018 [86] | Cerebral palsy patients after spinal arthrodesis | Mean 14 (SD 2.6) years | 212 | Prospective cohort study (NOS 3/9) | Pre-operative & 2 years | Parent: Caregiver Priorities and Child Health Index of Life with Disabilities (CPCHILD) | HRQoL scores significantly improved from preoperative (62) to 2-year follow-up (70) |
| Kyösti et al. 2018 [87] | Critically ill children admitted to the PICU | Mean 4.57 (SD 5.44) years | 1109 | Prospective cohort study (NOS 7/9) | 6 years | Parent: PedsQL  Child: 15D >16 years, 16D 12-15 years, 17D 8-11  years | 8.4% PedsQL scores <2 SD below the norm; low scores associated with elective admission, higher rate of neurological diagnoses, lower rate of respiratory diagnoses or sepsis than children with good QOL, 10.3% 15D-17D scores <2 SD below the norm |

^Scores for HRQoL questionnaires range from 0-100, unless otherwise indicated. For all questionnaires higher scores represent better performance.
Abbreviations: PICU, pediatric intensive care unit; IQR, interquartile range; ECMO, extracorporeal membrane oxygenation; RCT, randomized controlled trial; NOS, Newcastle Ottawa Scale; CCT, Cochrane Collaboration’s Tool; ARHQ, Agency for Healthcare Research and Quality; SD, standard deviation; SES, socioeconomic status^

**References**

1. Pollack, M. M., Holubkov, R., Funai, T., Clark, A., Berger, J. T., Meert, K., Newth, C. J., Shanley, T., Moler, F., Carcillo, J., Berg, R. A., Dalton, H., Wessel, D. L., Harrison, R. E., Doctor, A., Dean, J. M., Jenkins, T. L., Eunice Kennedy Shriver National Institute of Child, H., & Human Development Collaborative Pediatric Critical Care Research, N. (2014). Pediatric intensive care outcomes: development of new morbidities during pediatric critical care. Pediatr Crit Care Med, 15(9), 821-827.

2. Long, D. A., & Fink, E. L. (2021). Transitions from short to long-term outcomes in pediatric critical care: considerations for clinical practice. Transl Pediatr, 10(10), 2858-2874.

3. Atkins, E., Colville, G., & John, M. (2012). A 'biopsychosocial' model for recovery: a grounded theory study of families' journeys after a Paediatric Intensive Care Admission. Intensive Crit Care Nurs, 28(3), 133-140.

4. Benjafield, J. G. (2010). Cognition (4th edition ed.). Oxford: Oxford University Press

5. Kachmar, A. G., Irving, S. Y., Connolly, C. A., & Curley, M. A. Q. (2018). A Systematic Review of Risk Factors Associated With Cognitive Impairment After Pediatric Critical Illness. Pediatr Crit Care Med, 19(3), e164-e171.

6. Killien, E. Y., Loftis, L. L., Clark, J. D., Muszynski, J. A., Rissmiller, B. J., Singleton, M. N., White, B. R., Zimmerman, J. J., Maddux, A. B., Pinto, N. P., Fink, E. L., Watson, R. S., Smith, M., Ringwood, M., Graham, R. J., Post, P., Injury, P.-C. I. o. t. P. A. L., Sepsis, I., the Eunice Kennedy Shriver National Institute of Child, H., & Human Development Collaborative Pediatric Critical Care Research, N. (2021). Health-related quality of life outcome measures for children surviving critical care: a scoping review. Qual Life Res, 30(12), 3383-3394.

7. Procter, C., Morrow, B., Pienaar, G., Shelton, M., & Argent, A. (2021). Outcomes following admission to paediatric intensive care: A systematic review. J Paediatr Child Health, 57(3), 328-358.

8. Ko, M. S. M., Poh, P. F., Heng, K. Y. C., Sultana, R., Murphy, B., Ng, R. W. L., & Lee, J. H. (2022). Assessment of Long-term Psychological Outcomes After Pediatric Intensive Care Unit Admission: A Systematic Review and Meta-analysis. JAMA Pediatr, e215767.

9. Killien, E. Y., & Zimmerman, J. J. (2022). Long-term Psychological Morbidity Among Children Surviving Critical Illness and Injury. JAMA Pediatr, e215775.

10. Borenstein, M., Hedges, L.V., Higgins, J.P.T., Rothstein, H.R. (2009). Introduction to Meta-Analysis: John Wiley & Sons, Ltd.

11. Higgins, J. P. T., Thomas, J., Chandler, J., Cumpston, M., Li, T., Page, M. J., & Welch, V. A. (2021). Cochrane Handbook for Systematic Reviews of Interventions version 6.2 (updated February 2021). Cochrane, 2021. Available from www.training.cochrane.org/handbook.

12. Zeng, X., Zhang, Y., Kwong, J. S., Zhang, C., Li, S., Sun, F., Niu, Y., & Du, L. (2015). The methodological quality assessment tools for preclinical and clinical studies, systematic review and meta-analysis, and clinical practice guideline: a systematic review. J Evid Based Med, 8(1), 2-10.

13. Madderom, M. J., Schiller, R. M., Gischler, S. J., Van Heijst, A. F. J., Tibboel, D., Aarsen, F. K., & Ijsselstijn, H. (2016). Growing up after critical illness: Verbal, visual-spatial, and working memory problems in neonatal extracorporeal membrane oxygenation survivors. Crit Care Med, 44(6), 1182-1190.

14. Schiller, R. M., Madderom, M. J., van Rosmalen, J., van Heijst, A. F. J., de Blaauw, I., Utens, E., Rietman, A. B., Verhulst, F., Tibboel, D., White, T., & H, I. J. (2018). Working Memory Training Following Neonatal Critical Illness: A Randomized Controlled Trial. Crit Care Med, 46(7), 1158-1166.

15. Bembea, M. M., Felling, R. J., Caprarola, S. D., Ng, D. K., Tekes, A., Boyle, K., Yiu, A., Rizkalla, N., Schwartz, J., Everett, A. D., & Salorio, C. (2020). Neurologic Outcomes in a Two-Center Cohort of Neonatal and Pediatric Patients Supported on Extracorporeal Membrane Oxygenation. Asaio J., 66(1), 79-88.

16. Khalid, O. M., & Harrison, T. M. (2019). Early Neurodevelopmental Outcomes in Children with Hypoplastic Left Heart Syndrome and Related Anomalies After Hybrid Procedure. Pediatr Cardiol, 40(8), 1591-1598.

17. Meuwly, E., Feldmann, M., Knirsch, W., von Rhein, M., Payette, K., Dave, H., Tuura, R. O. G., Kottke, R., Hagmann, C., Latal, B., Jakab, A., Research Group, H., & Brain*. (2019). Postoperative brain volumes are associated with one-year neurodevelopmental outcome in children with severe congenital heart disease. Sci. rep., 9(1), 10885.

18. Purkayastha, J., Dutta, M., Lewis, L. E., Ramesh Bhat, Y., Joshua Rajan, X., & Ayappa, G. (2018). Study of neurodevelopmental outcomes at 10-14 months of age using bayley scale of infant and toddler development in asphyxiated newborns with hypoxic ischemic encephalopathy treated with and without therapeutic hypothermia. Iran J Neonatol, 9(4), 1-6.

19. Wei, X., Zhou, C., Ou, Y., & Qu, Q. (2019). Administration of surgical anesthetic dexmedetomidine improves cognitive and neurological function in infants with congenital heart disease. Int J Clin Exp Med, 12(6), 7527-7535.

20. Slomine, B. S., Nadkarni, V. M., Christensen, J. R., Silverstein, F. S., Telford, R., Topjian, A., Koch, J. D., Sweney, J., Fink, E. L., Mathur, M., Holubkov, R., Dean, J. M., & Moler, F. W. (2017). Pediatric cardiac arrest due to drowning and other respiratory etiologies: Neurobehavioral outcomes in initially comatose children. Resuscitation, 115, 178-184.

21. Slomine, B. S., Silverstein, F. S., Christensen, J. R., Page, K., Holubkov, R., Dean, J. M., & Moler, F. W. (2018). Neuropsychological Outcomes of Children 1 Year after Pediatric Cardiac Arrest: Secondary Analysis of 2 Randomized Clinical Trials. JAMA Neurol, 75(12), 1502-1510.

22. Slomine, B. S., Silverstein, F. S., Christensen, J. R., Holubkov, R., Telford, R., Dean, J. M., & Moler, F. W. (2018). Neurobehavioural outcomes in children after In-Hospital cardiac arrest. Resuscitation, 124, 80-89.

23. Slomine, B. S., Silverstein, F. S., Christensen, J. R., Holubkov, R., Page, K., Dean, J. M., & Moler, F. W. (2016). Neurobehavioral Outcomes in Children After Out-of-Hospital Cardiac Arrest. Pediatrics, 137(4).

24. Christensen, J. R., Slomine, B. S., Silverstein, F. S., Page, K., Holubkov, R., Dean, J. M., & Moler, F. W. (2019). Cardiac Arrest Outcomes in Children With Preexisting Neurobehavioral Impairment. Pediatr Crit Care Med, 20(6), 510-517.

25. Wilkinson, A. A., Simic, N., Frndova, H., Taylor, M. J., Choong, K., Fraser, D., Campbell, C., Dhanani, S., Kuehn, S., Beauchamp, M. H., Farrell, C., Anderson, V., Guerguerian, A. M., Dennis, M., Schachar, R., & Hutchison, J. S. (2016). Serum biomarkers help predict attention problems in critically ill children with traumatic brain injury. Pediatr Crit Care Med, 17(7), 638-648.

26. O'Brien, N. F., Maa, T., Moore-Clingenpeel, M., Rosenberg, N., & Yeates, K. O. (2018). Relationships between cerebral flow velocities and neurodevelopmental outcomes in children with moderate to severe traumatic brain injury. Childs Nerv. Syst., 34(4), 663-672.

27. Dennis, E. L., Ellis, M. U., Marion, S. D., Jin, Y., Moran, L., Olsen, A., Kernan, C., Babikian, T., Mink, R., Babbitt, C., Johnson, J., Giza, C. C., Thompson, P. M., & Asarnow, R. F. (2015). Callosal Function in Pediatric Traumatic Brain Injury Linked to Disrupted White Matter Integrity. J Neurosci, 35(28), 10202-10211.

28. Als, L. C., Tennant, A., Nadel, S., Cooper, M., Pierce, C. M., & Garralda, M. E. (2015). Persistence of neuropsychological deficits following pediatric critical illness. Crit Care Med, 43(8), e312-e315.

29. Kaur, J., Singhi, P., Singhi, S., Malhi, P., & Saini, A. G. (2015). Neurodevelopmental and behavioral outcomes in children with sepsis-associated encephalopathy admitted to pediatric intensive care unit: A prospective case control study. J Child Neurol, 31(6), 683-690.

30. Goldschmidt, I., van Dick, R., Jacobi, C., Junge, N., Pfister, E., Richter, N., & Baumann, U. (2019). Impact of Immunosuppression on Executive Functioning After Pediatric Liver Transplantation: An Observational Cohort Study. J Pediatr Gastroenterol Nutr, 68(4), 480-487.

31. Urschel, S., Bond, G. Y., Dinu, I. A., Moradi, F., Conway, J., Garcia-Guerra, G., Acton, B. V., Joffe, A. R., AlAklabi, M., Rebeyka, I. M., & Robertson, C. M. T. (2018). Neurocognitive outcomes after heart transplantation in early childhood. J Heart Lung Transplant, 37(6), 740-748.

32. Ryerson, L. M., Guerra, G. G., Joffe, A. R., Robertson, C. M. T., Alton, G. Y., Dinu, I. A., Granoski, D., Rebeyka, I. M., Ross, D. B., & Lequier, L. (2015). Survival and neurocognitive outcomes after cardiac extracorporeal life support in children less than 5 years of age: A ten-year cohort. Circ Heart Fail, 8(2), 312-321.

33. Varghese, A. D., Kulkarni, V., Kamath, S. P., Shenoy, R. D., & Shanthram Baliga, B. (2018). Cognitive functions after neonatal encephalopathy in a coastal city of South India-A retrospective cohort study. Indian J Public Health Res Dev, 9(11), 274-280.

34. Vassel-Hitier, J., Verdier, V., Rasquier, S., Chalard, A., Laurent-Vannier, A., & Chevignard, M. (2019). Language, intellectual and educational outcomes after moderate-to-severe traumatic brain injury sustained before the age of 18 months. Brain Inj, 33(8), 1105-1115.

35. Wagenaar, N., van den Berk, D. J. M., Lemmers, P. M. A., van der Aa, N. E., Dudink, J., van Bel, F., Groenendaal, F., de Vries, L. S., Benders, M. J. N. L., & Alderliesten, T. (2019). Brain Activity and Cerebral Oxygenation After Perinatal Arterial Ischemic Stroke Are Associated With Neurodevelopment. Stroke, 50(10), 2668-2676.

36. Leeuwen, L., Schiller, R. M., Rietman, A. B., Van Rosmalen, J., Wildschut, E. D., Houmes, R. J. M., Tibboel, D., & H, I. J. (2018). Risk factors of impaired neuropsychologic outcome in school-Aged survivors of neonatal critical illness. Crit Care Med, 46(3), 401-410.

37. Naguib, A. N., Winch, P. D., Tobias, J. D., Yeates, K. O., Miao, Y., Galantowicz, M., & Hoffman, T. M. (2015). Neurodevelopmental outcome after cardiac surgery utilizing cardiopulmonary bypass in children. Saudi J Anaesth, 9(1), 12-18.

38. Schiller, R. M., Madderom, M. J., Reuser, J. J. C. M., Steiner, K., Gischler, S. J., Tibboel, D., Van Heijst, A. F. J., & Ijsselstijn, H. (2016). Neuropsychological follow-up after neonatal ECMO. Pediatrics, 138(5).

39. Sterken, C., Lemiere, J., Van den Berghe, G., & Mesotten, D. (2016). Neurocognitive Development After Pediatric Heart Surgery. Pediatrics, 137(6).

40. Sadhwani, A., Cheng, H., Stopp, C., Rollins, C. K., Jolley, M. A., Dunbar-Masterson, C., Wypij, D., Newburger, J., Ware, J., & Thiagarajan, R. R. (2019). Early Neurodevelopmental Outcomes in Children Supported with ECMO for Cardiac Indications. Pediatr Cardiol, 40(5), 1072-1083.

41. Trieu, H. T., Anh, N. T. K., Vuong, H. N. T., Dao, T. T. M., Hoa, N. T. X., Tuong, V. N. C., Dinh, P. T., Wills, B., Qui, P. T., Van Tan, L., Yen, L. M., Sabanathan, S., & Thwaites, C. L. (2017). Long-term outcome in survivors of neonatal tetanus following specialist intensive care in Vietnam. BMC Infect Dis, 17(1).

42. Schiller, R. M., H, I. J., Madderom, M. J., Rietman, A. B., Smits, M., van Heijst, A. F. J., Tibboel, D., White, T., & Muetzel, R. L. (2017). Neurobiologic Correlates of Attention and Memory Deficits Following Critical Illness in Early Life. Crit Care Med.

43. Van Den Bosch, G. E., Ijsselstijn, H., Van Der Lugt, A., Tibboel, D., Van Dijk, M., & White, T. (2015). Neuroimaging, pain sensitivity, and neuropsychological functioning in school-age neonatal extracorporeal membrane oxygenation survivors exposed to opioids and sedatives. Pediatr Crit Care Med, 16(7), 652-662.

44. Schiller, R. M., H, I. J., Madderom, M. J., van Rosmalen, J., van Heijst, A. F. J., Smits, M., Verhulst, F., Tibboel, D., & White, T. (2019). Training-induced white matter microstructure changes in survivors of neonatal critical illness: A randomized controlled trial. Dev Cognitive Neurosci, 38.

45. Eismann, E. A., Theuerling, J., Cassedy, A., Curry, P. A., Colliers, T., & Makoroff, K. L. (2020). Early developmental, behavioral, and quality of life outcomes following abusive head trauma in infants. Child Abuse Negl, 108.

46. Schiller, R. M., van den Bosch, G. E., Muetzel, R. L., Smits, M., Dudink, J., Tibboel, D., Ijsselstijn, H., & White, T. (2017). Neonatal critical illness and development: white matter and hippocampus alterations in school-age neonatal extracorporeal membrane oxygenation survivors. Dev Med Child Neurol, 59(3), 304-310.

47. Volpe, D. S. J., Oliveira, N. C. A. C., Santos, A. C., Linhares, M. B. M., & Carlotti, A. P. C. P. (2017). Neuropsychological outcome of children with traumatic brain injury and its association with late magnetic resonance imaging findings: A cohort study. Brain Inj, 1-6.

48. Afshar, S., Porter, M., Barton, B., & Stormon, M. (2018). Intellectual and academic outcomes after pediatric liver transplantation: Relationship with transplant-related factors. Am J Transplant, 18(9), 2229-2237.

49. Eder, B., Melter, M., Gabler, V., Zant, R., & Knoppke, B. (2020). Risk factors associated with cognitive impairment in patients after pediatric liver transplantation. Pediatr Transplant.

50. Abend, N. S., Wagenman, K. L., Blake, T. P., Schultheis, M. T., Radcliffe, J., Berg, R. A., Topjian, A. A., & Dlugos, D. J. (2015). Electrographic status epilepticus and neurobehavioral outcomes in critically ill children. Epilepsy Behav, 49, 238-244.

51. Meyburg, J., Ries, M., Zielonka, M., Koch, K., Sander, A., von Haken, R., & Reuner, G. (2018). Cognitive and Behavioral Consequences of Pediatric Delirium: A Pilot Study. Pediatr Crit Care Med, 19(10), e531-e537.

52. Verstraete, S., Vanhorebeek, I., Covaci, A., Güiza, F., Malarvannan, G., Jorens, P. G., & Van den Berghe, G. (2016). Circulating phthalates during critical illness in children are associated with long-term attention deficit: a study of a development and a validation cohort. Intensive Care Med, 42(3), 379-392.

53. Verstraete, S., Verbruggen, S. C., Hordijk, J. A., Vanhorebeek, I., Dulfer, K., Güiza, F., van Puffelen, E., Jacobs, A., Leys, S., Durt, A., Van Cleemput, H., Eveleens, R. D., Garcia Guerra, G., Wouters, P. J., Joosten, K. F., & Van den Berghe, G. (2019). Long-term developmental effects of withholding parenteral nutrition for 1 week in the paediatric intensive care unit: a 2-year follow-up of the PEPaNIC international, randomised, controlled trial. Lancet Respir Med, 7(2), 141-153.

54. Jacobs, A., Dulfer, K., Eveleens, R. D., Hordijk, J., Van Cleemput, H., Verlinden, I., Wouters, P. J., Mebis, L., Guerra, G. G., Joosten, K., Verbruggen, S. C., Güiza, F., Vanhorebeek, I., & Van den Berghe, G. (2020). Long-term developmental effect of withholding parenteral nutrition in paediatric intensive care units: a 4-year follow-up of the PEPaNIC randomised controlled trial. Lancet Child Adolesc Health, 4(7), 503-514.

55. Meert, K., Slomine, B. S., Christensen, J. R., Telford, R., Holubkov, R., Dean, J. M., & Moler, F. W. (2018). Burden of caregiving after a child's in-hospital cardiac arrest. Resuscitation, 127, 44-50.

56. Vet, N. J., De Wildt, S. N., Verlaat, C. W. M., Mooij, M. G., Tibboel, D., De Hoog, M., & Buysse, C. M. P. (2016). Short-Term Health-Related Quality of Life of Critically Ill Children Following Daily Sedation Interruption∗. Pediatr Crit Care Med, 17(11), e513-e520.

57. Aspesberro, F., Fesinmeyer, M. D., Zhou, C., Zimmerman, J. J., & Mangione-Smith, R. (2016). Construct Validity and Responsiveness of the Pediatric Quality of Life Inventory 4.0 Generic Core Scales and Infant Scales in the PICU. Pediatr Crit Care Med, 17(6), e272-e279.

58. Choong, K., Fraser, D., Al-Harbi, S., Borham, A., Cameron, J., Cameron, S., Cheng, J., Clark, H., Doherty, T., Fayed, N., Gorter, J. W., Herridge, M., Khetani, M., Menon, K., Seabrook, J., Simpson, R., & Thabane, L. (2018). Functional Recovery in Critically Ill Children, the "weeCover" Multicenter Study. Pediatr Crit Care Med, 19(2), 145-154.

59. Colville, G. A., Pierce, C. M., & Peters, M. J. (2019). Self-Reported Fatigue in Children Following Intensive Care Treatment. Pediatr Crit Care Med, 20(2), e98-e101.

60. Hordijk, J., Verbruggen, S., Vanhorebeek, I., Van den Berghe, G., Utens, E., Joosten, K., & Dulfer, K. (2019). Health-related quality of life of children and their parents 6 months after children’s critical illness. Qual Life Res.

61. Ward, S. L., Turpin, A., Spicer, A. C., Treadwell, M. J., Church, G. D., & Flori, H. R. (2017). Long-Term Pulmonary Function and Quality of Life in Children After Acute Respiratory Distress Syndrome: A Feasibility Investigation. Pediatr Crit Care Med, 18(1), e48-e55.

62. Watson, R. S., Asaro, L. A., Hertzog, J. H., Sorce, L. R., Kachmar, A. G., Dervan, L. A., Angus, D. C., Wypij, D., & Curley, M. A. Q. (2018). Long-term outcomes after protocolized sedation versus usual care in ventilated pediatric patients. Am J Respir Crit Care Med, 197(11), 1457-1467.

63. Watson, R. S., Asaro, L. A., Hutchins, L., Bysani, G. K., Killien, E. Y., Angus, D. C., Wypij, D., & Curley, M. A. Q. (2019). Risk factors for functional decline and impaired quality of life after pediatric respiratory failure. Am J Respir Crit Care Med, 200(7), 900-909.

64. Fink, E. L., Beers, S. R., Houtrow, A. J., Richichi, R., Burns, C., Doughty, L., Ortiz-Aguayo, R., Madurski, C. A., Valenta, C., Chrisman, M., Golightly, L., Kiger, M., Patrick, C., Treble-Barna, A., Pollon, D., Smith, C. M., & Kochanek, P. (2019). Early Protocolized Versus Usual Care Rehabilitation for Pediatric Neurocritical Care Patients: A Randomized Controlled Trial. Pediatr Crit Care Med, 20(6), 540-550.

65. Ebrahim, S., & Parshuram, C. (2015). Comparison of utility scores from the Visual Analog Scale and Health Utilities Index 3 in children following pediatric intensive care unit admission. J Child Health Care, 19(1), 53-62.

66. Peña, R., Suman, O. E., Rosenberg, M., Andersen, C. R., Herndon, D. N., & Meyer, W. J. (2018). One-Year Comparison of a Community-Based Exercise Program Versus a Day Hospital-Based Exercise Program on Quality of Life and Mental Health in Severely Burned Children. Arch Phys Med Rehabil.

67. Biagas, K. V., Hinton, V. J., Hasbani, N. R., Luckett, P. M., Wypij, D., Nadkarni, V. M., Agus, M. S. D., Srinivasan, V., Mourani, P. M., Chima, R., Thomas, N. J., Li, S., Pinto, A., Newth, C., Hassinger, A., Bysani, K., Rehder, K. J., Faustino, E. V., Kandil, S., Hirshberg, E., Wintergerst, K., Schwarz, A., Bagdure, D., Marsillio, L., Cvijanovich, N., Pham, N., Quasney, M., Flori, H., Federman, M., Nett, S., Pinto, N., Viteri, S., Schneider, J., Medar, S., Sapru, A., McQuillen, P., Babbitt, C., Lin, J. C., Jouvet, P., Yanay, O., Allen, C., Asaro, L., Coughlin-Wells, K., French, J., & Natarajan, A. (2020). Long-Term Neurobehavioral and Quality of Life Outcomes of Critically Ill Children after Glycemic Control. J Pediatr, 218, 57-63.e55.

68. Boeschoten, S. A., Dulfer, K., Boehmer, A. L. M., Merkus, P. J. F. M., van Rosmalen, J., de Jongste, J. C., de Hoog, M., Buysse, C. M. P., Heisterkamp, S., van Woensel, J., Haarman, E., Kapitein, B., Wösten-van Asperen, R., Kneyber, M., Lemson, J., Hartman, S., van Waardenburg, D., Bunker, H., Brouwer, C., van Ewijk, B., Landstra, A., Verwaal, M., Vaessen-Verberne, A., & Hammer, S. (2020). Quality of life and psychosocial outcomes in children with severe acute asthma and their parents. Pediatr Pulmonol, 55(11), 2883-2892.

69. Meert, K. L., Reeder, R., Maddux, A. B., Banks, R., Berg, R. A., Zuppa, A., Newth, C. J., Wessel, D., Pollack, M. M., Hall, M. W., Quasney, M., Sapru, A., Carcillo, J. A., McQuillen, P. S., Mourani, P. M., Chima, R. S., Holubkov, R., Sorenson, S., Varni, J. W., McGalliard, J., Haaland, W., Whitlock, K. B., Dean, J. M., & Zimmerman, J. J. (2020). Trajectories and Risk Factors for Altered Physical and Psychosocial Health-Related Quality of Life after Pediatric Community-Acquired Septic Shock∗. Pediatr Crit Care Med, 869-878.

70. Meert, K. L., Reeder, R. W., Maddux, A. B., Banks, R., Berg, R. A., Newth, C. J., Hall, M. W., Quasney, M., Carcillo, J. A., McQuillen, P. S., Mourani, P. M., Chima, R. S., Holubkov, R., Sorenson, S., McGalliard, J., Dean, J. M., & Zimmerman, J. J. (2020). Health-Related Quality of Life After Community-Acquired Septic Shock in Children With Preexisting Severe Developmental Disabilities. Pediatr Crit Care Med.

71. Nelson, L. P., Lachman, S. E., Goodman, K., & Gold, J. I. (2020). Admission Psychosocial Characteristics of Critically Ill Children and Acute Stress. Pediatr Crit Care Med.

72. Silver, G., Doyle, H., Hegel, E., Kaur, S., Mauer, E. A., Gerber, L. M., & Traube, C. (2020). Association Between Pediatric Delirium and Quality of Life After Discharge. Crit Care Med, 48(12), 1829-1834.

73. Zimmerman, J. J., Banks, R., Berg, R. A., Zuppa, A., Newth, C. J., Wessel, D., Pollack, M. M., Meert, K. L., Hall, M. W., Quasney, M., Sapru, A., Carcillo, J. A., McQuillen, P. S., Mourani, P. M., Wong, H., Chima, R. S., Holubkov, R., Coleman, W., Sorenson, S., Varni, J. W., McGalliard, J., Haaland, W., Whitlock, K., Michael Dean, J., Reeder, R. W., Meert, K. L., Heidemann, S., Pawluszka, A., Lulic, M., Berg, R. A., Zuppa, A., Twelves, C., DiLiberto, M. A., Pollack, M., Wessel, D., Berger, J., Tomanio, E., Hession, D., Wolfe, A., Mourani, P., Carpenter, T., Ladell, D., Sierra, Y., Rutebemberwa, A., Hall, M., Yates, A., Steele, L., Flowers, M., Hensley, J., Sapru, A., Harrison, R., Ashtari, N., Ratiu, A., Carcillo, J., Bell, M., Koch, L., Abraham, A., McQuillen, P., McKenzie, A., Zetino, Y., Newth, C., Kwok, J., Yamakawa, A., Quasney, M., Shanley, T., Jay-Achandran, C. J., Chima, R., Wong, H., Krallman, K., Stoneman, E., Benken, L., Yunger, T., Doctor, A., Eaton, M., Zimmerman, J. J., Chen, C., Sullivan, E., Merritt, C., Rich, D., McGalliard, J., Haaland, W., Whitlock, K., Salud, D., Michael Dean, J., Holubkov, R., Coleman, W., Sorenson, S., Reeder, R., Banks, R., Webster, A., Burr, J., Bisping, S., Liu, T., Stock, E., Flick, K., & Varni, J. (2020). Critical illness factors associated with long-term mortality and health-related quality of life morbidity following community-acquired pediatric septic shock. Crit Care Med, 319-328.

74. Zimmerman, J. J., Banks, R., Berg, R. A., Zuppa, A., Newth, C. J., Wessel, D., Pollack, M. M., Meert, K. L., Hall, M. W., Quasney, M., Sapru, A., Carcillo, J. A., McQuillen, P. S., Mourani, P. M., Wong, H., Chima, R. S., Holubkov, R., Coleman, W., Sorenson, S., Varni, J. W., McGalliard, J., Haaland, W., Whitlock, K., Michael Dean, J., Reeder, R. W., Meert, K. L., Heidemann, S., Pawluszka, A., Lulic, M., Berg, R. A., Zuppa, A., Twelves, C., DiLiberto, M. A., Pollack, M., Wessel, D., Berger, J., Tomanio, E., Hession, D., Wolfe, A., Mourani, P., Carpenter, T., Ladell, D., Sierra, Y., Rutebemberwa, A., Hall, M., Yates, A., Steele, L., Flowers, M., Hensley, J., Sapru, A., Harrison, R., Ashtari, N., Ratiu, A., Carcillo, J., Bell, M., Koch, L., Abraham, A., McQuillen, P., McKenzie, A., Zetino, Y., Newth, C., Kwok, J., Yamakawa, A., Quasney, M., Shanley, T., Jayachandran, C. J., Chima, R., Wong, H., Krallman, K., Stoneman, E., Benken, L., Yunger, T., Doctor, A., Eaton, M., Zimmerman, J. J., Chen, C., Sullivan, E., Merritt, C., Rich, D., McGalliard, J., Haaland, W., Whitlock, K., Salud, D., Michael Dean, J., Holubkov, R., Coleman, W., Sorenson, S., Reeder, R., Banks, R., Webster, A., Burr, J., Bisping, S., Liu, T., Stock, E., Flick, K., & Varni, J. (2020). Trajectory of mortality and health-related quality of life morbidity following community-acquired pediatric septic shock. Crit Care Med, 329-337.

75. Campbell, H., Eddama, O., Azzopardi, D., Edwards, A. D., Strohm, B., & Rivero-Arias, O. (2018). Hypothermia for perinatal asphyxia: Trial-based quality of life at 6-7 years. Arch Dis Child, 103(7), 654-659.

76. Di Leo, V., Biban, P., Mercolini, F., Martinolli, F., Pettenazzo, A., Perilongo, G., & Amigoni, A. (2019). The quality of life in extracorporeal life support survivors: single-center experience of a long-term follow-up. Child's Nerv Syst, 35(2), 227-235.

77. Elias, M. D., Achuff, B. J., Ittenbach, R. F., Ravishankar, C., Spray, T. L., Fuller, S., Montenegro, L. M., Gaynor, J. W., & O’Connor, M. J. (2017). Long-Term Outcomes of Pediatric Cardiac Patients Supported by Extracorporeal Membrane Oxygenation. Pediatr Crit Care Med.

78. Fleck, T. P. K., Dangel, G., Bächle, F., Benk, C., Grohmann, J., Kroll, J., Siepe, M., Höhn, R., Kirschner, J., Beyersdorf, F., & Stiller, B. (2017). Long-Term Follow-Up on Health-Related Quality of Life after Mechanical Circulatory Support in Children. Pediatr Crit Care Med, 18(2), 176-182.

79. Öst, E., Frenckner, B., Nisell, M., Burgos, C. M., & Öjmyr-Joelsson, M. (2018). Health-related quality of life in children born with congenital diaphragmatic hernia. Pediatr Surg Int, 34(4), 405-414.

80. van Zellem, L., Utens, E. M., Legerstee, J. S., Cransberg, K., Hulst, J. M., Tibboel, D., & Buysse, C. (2015). Cardiac Arrest in Children: Long-Term Health Status and Health-Related Quality of Life. Pediatr Crit Care Med, 16(8), 693-702.

81. Yu, Y. R., Carpenter, J. L., DeMello, A. S., Keswani, S. G., Cass, D. L., Olutoye, O. O., Vogel, A. M., Thomas, J. A., Burgman, C., Fernandes, C. J., & Lee, T. C. (2018). Evaluating quality of life of extracorporeal membrane oxygenation survivors using the pediatric quality of life inventory survey. J Pediatr Surg, 53(5), 1060-1064.

82. Polić, B., Bubić, A., Meštrović, J., Markić, J., Kovačević, T., Furlan, I. A., Utrobičić, I., & Kolčić, I. (2017). Emotional and behavioral outcomes and quality of life in school-age children born as late preterm: Retrospective cohort study. Croat Med J, 58(5), 332-341.

83. Hordijk, J., Verbruggen, S., Vanhorebeek, I., Güiza, F., Wouters, P., Van Den Berghe, G., Joosten, K., & Dulfer, K. (2020). Health-related quality of life of children and their parents 2 years after critical illness: pre-planned follow-up of the PEPaNIC international, randomized, controlled trial. Crit Care, 24(1).

84. Chandler, H. K., Teppa, B., Johnson, K. A., McCracken, C., Fortenberry, J. D., & Paden, M. L. (2015). Determining comorbidities and quality of life among pediatric survivors of extracorporeal life support. J Crit Care, 30(5), 1085-1089.

85. Ekinci, O., Okuyaz, Ç., Günes, S., Ekinci, N., Örekeci, G., Teke, H., & Çobanoǧullarl Direk, M. (2017). Sleep and quality of life in children with traumatic brain injury and ADHD: A comparison with primary ADHD. Int J Psychiatry Med, 52(1), 72-87.

86. Jain, A., Sullivan, B. T., Shah, S. A., Samdani, A. F., Yaszay, B., Marks, M. C., & Sponseller, P. D. (2018). Caregiver perceptions and health-related quality-of-life changes in cerebral palsy patients after spinal arthrodesis. Spine, 43(15), 1052-1056.

87. Kyösti, E., Ala-Kokko, T. I., Ohtonen, P., Peltoniemi, O., Rautiainen, P., Kataja, J., Ebeling, H., & Liisanantti, J. H. (2018). Factors associated with health-related quality of life 6 years after ICU discharge in a Finnish paediatric population: a cohort study. Intensive Care Med, 44(9), 1378-1387.

88. Germain, N., Aballea, S., & Toumi, M. (2019). Measuring the health-related quality of life in young children: how far have we come? J Mark Access Health Policy, 7(1), 1618661.

89. Herrup, E. A., Wieczorek, B., & Kudchadkar, S. R. (2017). Characteristics of postintensive care syndrome in survivors of pediatric critical illness: A systematic review. World J Crit Care Med, 6(2), 124-134.

90. Rennick, J. E., & Childerhose, J. E. (2015). Redefining success in the PICU: new patient populations shift targets of care. Pediatrics, 135(2), e289-291.

91. Burns, J. P., Sellers, D. E., Meyer, E. C., Lewis-Newby, M., & Truog, R. D. (2014). Epidemiology of death in the PICU at five U.S. teaching hospitals*. Crit Care Med, 42(9), 2101-2108.

92. Lopes-Junior, L. C., Rosa, M., & Lima, R. A. G. (2018). Psychological and Psychiatric Outcomes Following PICU Admission: A Systematic Review of Cohort Studies. Pediatr Crit Care Med, 19(1), e58-e67.

93. Baker, S. C., & Gledhill, J. A. (2017). Systematic Review of Interventions to Reduce Psychiatric Morbidity in Parents and Children After PICU Admissions. Pediatr Crit Care Med, 18(4), 343-348.

94. Colville, G., & Pierce, C. (2012). Patterns of post-traumatic stress symptoms in families after paediatric intensive care. Intensive Care Med, 38(9), 1523-1531.

95. Lee, K., Cascella, M., & Marwaha, R. (2020). Intellectual Disability.

96. Jurrius K, B. K., Goes I, Hofstra-Windesheim A. . (2016). Niet-aangeboren hersenletsel: oorzaken, gevolgen, signalen en zorg Basisinformatie over niet-aangeboren hersenletsel voor (aankomende) wijkprofes sionals en studenten in de zorg en hulpverlening. Almere: Windesheim Flevoland.

97. Eiser, C., & Varni, J. W. (2013). Health-related quality of life and symptom reporting: similarities and differences between children and their parents. Eur J Pediatr, 172(10), 1299-1304.

98. Manning, J. C., Pinto, N. P., Rennick, J. E., Colville, G., & Curley, M. A. Q. (2018). Conceptualizing Post Intensive Care Syndrome in Children-The PICS-p Framework. Pediatr Crit Care Med, 19(4), 298-300.

99. Knoester, H., Grootenhuis, M. A., & Bos, A. P. (2007). Outcome of paediatric intensive care survivors. Eur J Pediatr, 166(11), 1119-1128.

100. Maddux, A. B., Pinto, N., Fink, E. L., Hartman, M. E., Nett, S., Biagas, K., Killien, E. Y., Dervan, L. A., Christie, L. M., Luckett, P. M., Loftis, L., Lackey, M., Ringwood, M., Smith, M., Olson, L., Sorenson, S., Meert, K. L., Notterman, D. A., Pollack, M. M., Mourani, P. M., Watson, R. S., Pediatric Outcomes, S. a. P., Injury, P.-C. I. o. t. P. A. L., Sepsis, I., the Eunice Kennedy Shriver National Institute of Child, H., & Human Development Collaborative Pediatric Critical Care Research, N. (2020). Postdischarge Outcome Domains in Pediatric Critical Care and the Instruments Used to Evaluate Them: A Scoping Review. Crit Care Med, 48(12), e1313-e1321.

101. Halpern, N. (2019). Critical Care Statistics. Retrieved May 1, 2020, from https://www.sccm.org/Communications/Critical-Care-Statistics
